# Recurrent *De Novo* NAHR Reciprocal Duplications in the *ATAD3* Gene Cluster Cause a Neurogenetic Trait with Perturbed Cholesterol and Mitochondrial Metabolism

**DOI:** 10.1016/j.ajhg.2020.01.007

**Published:** 2020-01-30

**Authors:** Adam C. Gunning, Klaudia Strucinska, Mikel Muñoz Oreja, Andrew Parrish, Richard Caswell, Karen L. Stals, Romina Durigon, Karina Durlacher-Betzer, Mitchell H. Cunningham, Christopher M. Grochowski, Julia Baptista, Carolyn Tysoe, Emma Baple, Nayana Lahiri, Tessa Homfray, Ingrid Scurr, Catherine Armstrong, John Dean, Uxoa Fernandez Pelayo, Aleck W.E. Jones, Robert W. Taylor, Vinod K. Misra, Wan Hee Yoon, Caroline F. Wright, James R. Lupski, Antonella Spinazzola, Tamar Harel, Ian J. Holt, Sian Ellard

**Affiliations:** 1 Exeter Genomics Laboratory, Royal Devon and Exeter NHS Foundation Trust, Exeter EX2 5DW, UK; 2 Institute of Biomedical and Clinical Science, College of Medicine and Health, University of Exeter, Exeter EX2 5DW, UK; 3 Aging and Metabolism Research Program, Oklahoma Medical Research Foundation, Oklahoma City, OK 73104, USA; 4 Biodonostia Health Research Institute, 20014 San Sebastián, Spain; 5 Department of Clinical and Movement Neurosciences, UCL Queen Square Institute of Neurology, Royal Free Campus, London NW3 2PF, UK; 6 Department of Genetic and Metabolic Diseases, Hadassah-Hebrew University Medical Center, Jerusalem 91120, Israel; 7 Department of Pediatrics, Division of Genetic, Genomic, and Metabolic Disorders, Wayne State University School of Medicine, Children’s Hospital of Michigan, Detroit, MI 48201, USA; 8 Department of Molecular and Human Genetics, Baylor College of Medicine, Houston, TX 77030, USA; 9 South West Thames Regional Genetics Service, St George’s University Hospitals NHS Foundation Trust, London SW17 0QT, UK; 10 St George’s University of London, London SW17 0RE, UK; 11 Department of Clinical Genetics, University Hospitals Bristol NHS Foundation Trust, Bristol BS2 8EG, UK; 12 Department of Paediatric Cardiology, University Hospitals Bristol NHS Foundation Trust, Bristol BS2 8BJ, UK; 13 Clinical Genetics Service, NHS Grampian, Aberdeen Royal Infirmary, Aberdeen AB25 2ZA, UK; 14 Wellcome Centre for Mitochondrial Research, Translational and Clinical Research Institute, Faculty of Medical Sciences, Newcastle University, Newcastle upon Tyne NE2 4HH, UK; 15 Department of Pediatrics, Baylor College of Medicine, Houston, TX 77030, USA; 16 Human Genome Sequencing Center, Baylor College of Medicine, Houston, TX 77030, USA; 17 Texas Children’s Hospital, Houston, TX 77030, USA; 18 MRC Centre for Neuromuscular Diseases, UCL Queen Square Institute of Neurology and National Hospital for Neurology and Neurosurgery, Queen Square, London WC1N 3BG, UK; 19 IKERBASQUE, Basque Foundation for Science, 48013 Bilbao, Spain; 20 CIBERNED (Center for Networked Biomedical Research on Neurodegenerative Diseases, Ministry of Economy and Competitiveness, Institute Carlos III), 28031 Madrid, Spain

**Keywords:** mitochondrial DNA, cholesterol, cardiomyopathy, ATAD3, ATAD3 gene cluster, NAHR, non-allelic homologous recombination, metabolic disorder, Harel-Yoon

## Abstract

Recent studies have identified both recessive and dominant forms of mitochondrial disease that result from *ATAD3A* variants. The recessive form includes subjects with biallelic deletions mediated by non-allelic homologous recombination. We report five unrelated neonates with a lethal metabolic disorder characterized by cardiomyopathy, corneal opacities, encephalopathy, hypotonia, and seizures in whom a monoallelic reciprocal duplication at the *ATAD3* locus was identified. Analysis of the breakpoint junction fragment indicated that these 67 kb heterozygous duplications were likely mediated by non-allelic homologous recombination at regions of high sequence identity in *ATAD3A* exon 11 and *ATAD3C* exon 7. At the recombinant junction, the duplication allele produces a fusion gene derived from *ATAD3A* and *ATAD3C*, the protein product of which lacks key functional residues. Analysis of fibroblasts derived from two affected individuals shows that the fusion gene product is expressed and stable. These cells display perturbed cholesterol and mitochondrial DNA organization similar to that observed for individuals with severe ATAD3A deficiency. We hypothesize that the fusion protein acts through a dominant-negative mechanism to cause this fatal mitochondrial disorder. Our data delineate a molecular diagnosis for this disorder, extend the clinical spectrum associated with structural variation at the *ATAD3* locus, and identify a third mutational mechanism for *ATAD3* gene cluster variants. These results further affirm structural variant mutagenesis mechanisms in sporadic disease traits, emphasize the importance of copy number analysis in molecular genomic diagnosis, and highlight some of the challenges of detecting and interpreting clinically relevant rare gene rearrangements from next-generation sequencing data.

## Main Text

Since its initial association with a neurological disorder,^1^ it has become apparent that disruption of the *ATAD3* cluster, and more specifically *ATAD3A* (MIM: 612316), is a significant cause of pediatric disease. Variants at this locus are associated with a wide phenotypic spectrum, including pontocerebellar hypoplasia,^2^ hereditary spastic paraplegia,^3^ and a syndromic neurological disorder characterized by peripheral neuropathy, hypotonia, cardiomyopathy, optic atrophy, cerebellar atrophy, and seizures:^1^ Harel-Yoon syndrome (HAYOS [MIM: 617183]). The different phenotypes can be attributed to a spectrum of disease-causing variants that includes bi-allelic hypomorphic variants, bi-allelic deletions, and monoallelic dominant-negative missense variants. Here, we report two *de novo* intergenic duplications in the *ATAD3* cluster identified in five unrelated neonates with shared phenotypes including corneal clouding, cardiomyopathy, hypotonia, and white matter changes, thus expanding the genotype spectrum of *ATAD3*-related disorders.

The *ATAD3* cluster is composed of three paralogs with extensive sequence homology, formed through tandem segmental duplication: *ATAD3A*, *ATAD3B* (MIM: 612317), and *ATAD3C* (MIM: 617227). *ATAD3A* and *ATAD3B* are protein-coding genes of near identical sequence, differing primarily due to a stop-loss mutation in *ATAD3B* that extends the protein by 62 amino acids; *ATAD3C* is not known to be expressed. ATAD3A is a transmembrane ATPase, which is predicted to form hexamers,^4^ a fraction of which is found at contact sites between the inner and outer mitochondrial membranes^5^ in complex with TSPO, CYP11A1, and OPA1.^6^ ATAD3 has also been shown to interact with mitochondrial nucleoprotein complexes and to play roles in mtDNA organization and replication.^2^^,^^7^^,^^8^ More recently it has been shown to interact with Drp1/DNM1L to support Drp1-induced mitochondrial division,^9^ a process that drives mtDNA segregation.^10^^,^^11^ Concordantly, ATAD3 dysfunction and deficiency have a wide range of effects on mitochondrial structure and function, characterized by disturbed mitochondrial morphology and fission dynamics,^3^^,^^6^ loss of cristae,^12^ perturbed mtDNA and cholesterol metabolism, impaired mitochondrial steroidogenesis,^2^^,^^13^ and decreased levels of some mitochondrial oxidative phosphorylation (OXPHOS) components.^12^ It is not clear whether the disruption to the inner mitochondrial membrane, mtDNA, and OXPHOS complexes are due directly to the absence of ATAD3^4^^,^^12^ or whether they are consequences of changes to membrane architecture resulting from an altered cholesterol content^2^^,^^13^ or a combination of the two.

**Figure 1:**
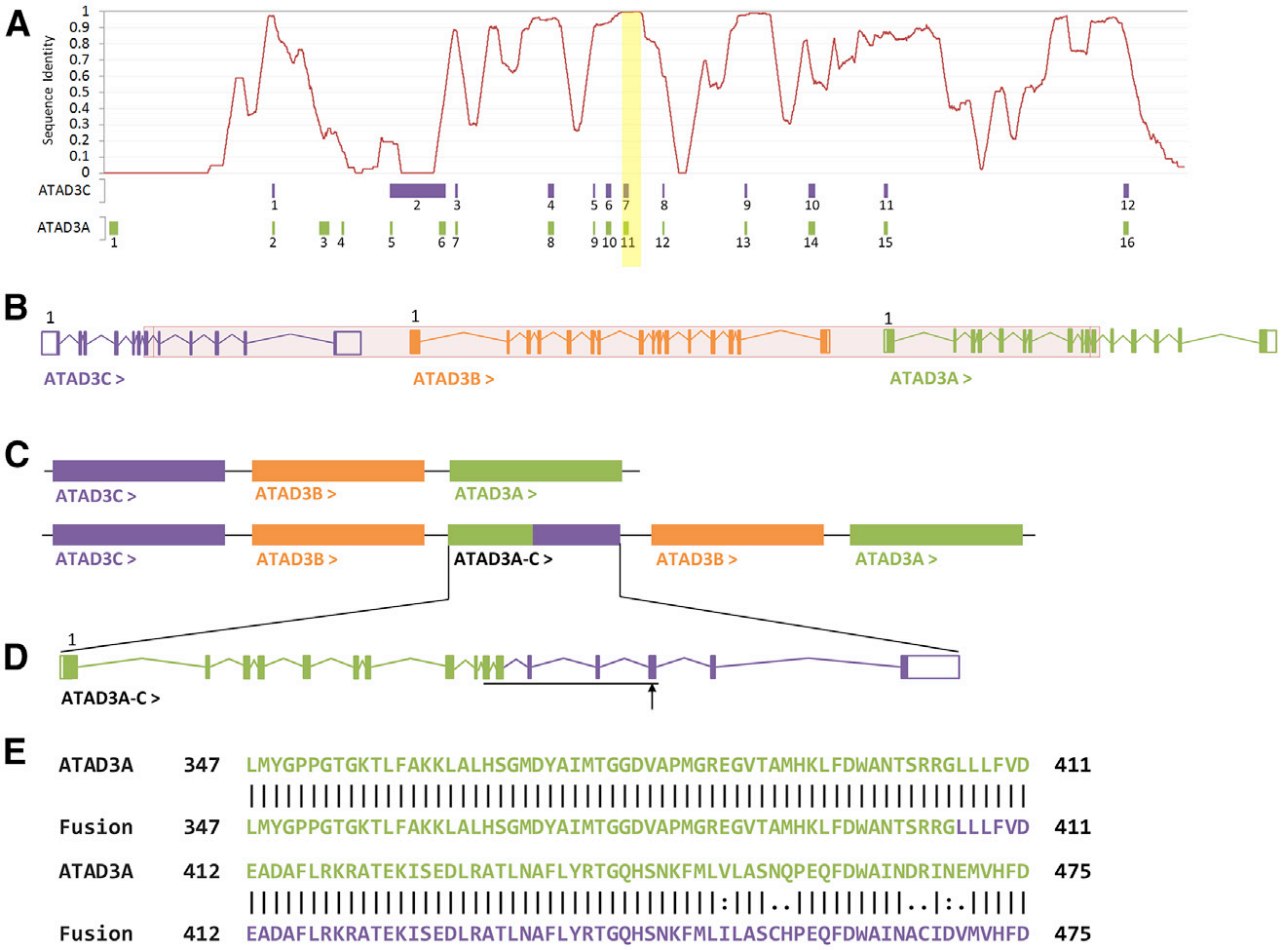
NAHR between *ATAD3C* Exon 8 and *ATAD3A* Exon 11 Produces a Fusion Gene, with Variants at Key Functional Residues within the ATPase Domain Gene intron-exon structures are shown in cartoon format; open boxes indicate UTRs while closed boxes indicate coding regions. Arrows following the gene name indicate reading direction, and the first exon is labeled. Genes are shown in their relative position on chromosome 1 in a 5′ to 3′ direction from left to right. (A) Nucleotide sequence identity between *ATAD3A* (chr1:1512151–1534687:1) and *ATAD3*C (chr1:1449689–1470158:1) in a sliding 500 bp window. *ATAD3A* and *ATAD3C* exon positions are represented below according to their relative position within the KAlign alignment; this includes alignment gaps. The 398 bp region of 100% sequence identity is marked in yellow. (B) Reference arrangement of the ATAD3 cluster showing the exon structures of *ATAD3C* (purple), *ATAD3B* (orange), and *ATAD3A* (green). The duplicated region is highlighted in red. (C) The reference arrangement of the *ATAD3* cluster above the predicted configuration following duplication. (D) The exon structure of the *ATAD3A-C* fusion gene, with exons 1–11 derived from ATAD3A (green) and exons 12–16 derived from *ATAD3C* (purple). The ATPase domain is underlined (Asn347-Leu475; PFam PF00004), with the position of a key functional residue, Arg466, indicated by an arrow. (E) Amino acid sequence of the ATPase domain of ATAD3A (top) and the predicted amino acids sequence of the ATAD3A-C fusion protein (bottom). The green residues are derived from *ATAD3A*, while the purple residues are derived from *ATAD3C*. A vertical bar (|) indicates an identical amino acid, a colon (:) indicates a strongly conservative amino acid change (score > 0.5 in PAM250 matrix), and a period (.) indicates a weakly conservative amino acid change (score = < 0.5 in PAM250 matrix). The sequences differ at seven positions.

We report *de novo ATAD3* duplications identified in five unrelated neonates through exome sequencing. Clinical exome sequencing failed to identify any alternative molecular diagnosis potentially causative of the phenotype, which is characterized by seizures (four of the five neonates) and fetal akinesia and contractures (in three case subjects). A clinical summary is shown in Table 1 and clinical case reports are detailed in the Supplemental Note. Informed consent was obtained and all processes adhered to local and national ethical standards. The duplication in the *ATAD3* cluster was also detected by arrayCGH for those subjects studied (subjects four and five). The duplication is predicted to be the product of non-alleleic homologous recombination (NAHR) between regions of high sequence homology in *ATAD3C* and *ATAD3A* (Figure 1A) and encompasses *ATAD3C* exons 8–12, *ATAD3B*, and *ATAD3A* exons 1–11 (Figures 1B, S1, and S2).

**Table 1:** Clinical Features of Individuals with Duplication in *ATAD3* Gene Cluster

	**Subject 1**	**Subject 2**	**Subject 3**	**Subject 4**	**Subject 5**
Sex	male	female	male	female	male
Gestation	term	38 weeks	term	33+3 weeks	term
Apgars at birth	3	poor	1	5,8,9	1,0
Chronological age at death	3 days	6 weeks	5 days	6 weeks	4 weeks
Cardiomyopathy	HCM	DCM	DCM; cardiomegaly	HCM; cardiomegaly	HCM; cardiomegaly
Congenital cataracts	✓	ND	ND	ND	ND
Corneal opacity	✓	✓	✓	✓	✓
Postnatal hypotonia	✓	✓	✓	✓	✓
Abnormality of the external genitalia	cryptorchidism and micropenis	ND	ND	ND	hypospadias
Seizures	✓	diffuse abnormalities on EEG	ND	diffuse abnormalities on EEG	✓
Encephalopathy	✓	ND	✓	ND	ND
Brain findings	ND	white matter changes; simplified gyral patterning; cerebellar atrophy (MRI)	widespread hypoxic brain damage (post-mortem)	diffuse bilateral abnormal subcortical, periventricular, and deep white matter; abnormal MR spectroscopy	white matter changes, generalized reduction of brain volume (MRI); abnormal MR spectroscopy (lactate peak) on day 9
Contractures/fetal akinesia	fetal akinesia	ND	contractures	ND	contractures
Edema/fetal hydrops	ND	fetal hydrops; edema	fetal hydrops	ND	ND
Metabolic investigations	increased excretion of fumarate, malate, 2-ketoglutarate, 3-methylglutaconate, and 3-methylglutarate	lactic acidosis	ND	lactic acidosis; increased excretion of 2OH butyrate, fumarate, and 3OH isobutyrate	lactic acidosis, increased excretion of fumarate, malate on day 22
Prior genetic investigations	ArrayCGH; Prader-Willi; SMA	prenatal aneuploidy	ND	arrayCGH; 202 gene mitochondrial panel	arrayCGH, 27 gene glycogen storage disease panel

HCM, hypertrophic cardiomyopathy; DCM, dilated cardiomyopathy; SMA, spinal muscular atrophy; ND, not detected.

PCR and Sanger sequencing confirmed the presence of the duplications, which showed a 1.2 kb proband-specific amplicon (1.6 kb for subject four due to alternative primer design; data not shown). No PCR product was amplified in DNA derived from unaffected parents, consistent with a *de novo* event, and proband-parent relationships were confirmed for all case subjects during exome analysis. The 5′ end of the PCR amplicon was derived from *ATAD3A* exon 10, while the 3′ was derived from *ATAD3C* intron 7. The breakpoints of the duplication identified in subject four were found to differ from those identified in the other case subjects. The duplications are considered functionally equivalent as their protein products are predicted to be identical, differing at a single intronic nucleotide. These results are consistent with tandem duplication without inversion, described as NC_000001.11(GRCh38):1456616_1524663dup (subjects 1–3 and 5) and NC_000001.11(GRCh38):1456890_1524937dup (subject 4). The duplications are predicted to maintain the copy-number of *ATAD3A* and *ATAD3C*, duplicate *ATAD3B*, and create a fusion gene, *ATAD3A-C*, composed of *ATAD3A* (Uniprot: Q9NVI7-2, residues 1–405) and *ATAD3C* (Uniprot: Q5T2N8-1, residues 231–411) (Figures 1B and 1C).

We performed multiple complementary *in silico* analyses to characterize the effect of the duplication. Multiple sequence alignment of *ATAD3A* (NC_000001.11(GRCh38):1512151–1534687) and *ATAD3C* (NC_000001.11(GRCh38):1449689–1470158) showed the genes have an overall sequence identity of approximately 56%. The duplications occur at a 673 bp region with near-complete sequence identity between *ATAD3A* and *ATAD3C* (Figure 1A). *In silico* splicing analysis of *ATAD3A-C* showed that the splice sites are maintained (Figure S3). Pairwise alignment of ATAD3A (GenBank: NM_001170535.2; Q9NVI7-2) and ATAD3A-C (Uniprot: Q9NVI7-2, residues 1–405, and Uniprot: Q5T2N8-1, residues 231–411) primary amino acid sequences showed that they are of identical length and differ at 29 residues (Figure S4). Seven of the variants (p.Val450Ile, p.Asn454Cys, p.Gln455His, p.Asp465Ala, p.Arg466Cys, p.Asn468Asp, and p.Glu469Val) lie within the ATPase domain (residues 348–474; Pfam: PF00004) (Figure 1D, underline; Figure 1E), while the remaining 22 are present outside of a known functional domain (p.Glu482Ala, p.Phe489Leu, p.Asp490Asn, p.Lys491Glu, p.Gln502Arg, p.Ser516Leu, p.Val518Ile, p.Gly527Cys, p.Glu529Lys, and p.Glu545Lys) or within a region of predicted intrinsic disorder (p.Thr556Ala, p.Arg557Cys, p.Ala561Phe, p.Lys568Met, p.Cys570Arg, p.Ala574Gly, p.Gly576Arg, p.Arg579Pro, p.Gly580Glu, p.Pro583Gln, p.Ser584Pro, and p.Pro585Ser). DeepLoc (v1.0) was used to predict the subcellular localization of ATAD3A and ATAD3A-C. The tool was able to correctly predict that ATAD3A is transported into the mitochondrial membrane. There was no change in this prediction for ATAD3A-C. Together, these analyses indicate that the fusion transcript is likely to be correctly transcribed and translated and maintain the signals necessary for native subcellular localization. We next modeled the composition of ATAD3 hexamers using a binomial distribution based on two copies of *ATAD3A* and one copy of *ATAD3A-C*. It is predicted that 8.8% of ATAD3 hexamers would be comprised solely of wild-type ATAD3A monomers, while 91.2% would contain at least one copy of the ATAD3A-C fusion protein (Figure S5).

**Figure 2:**
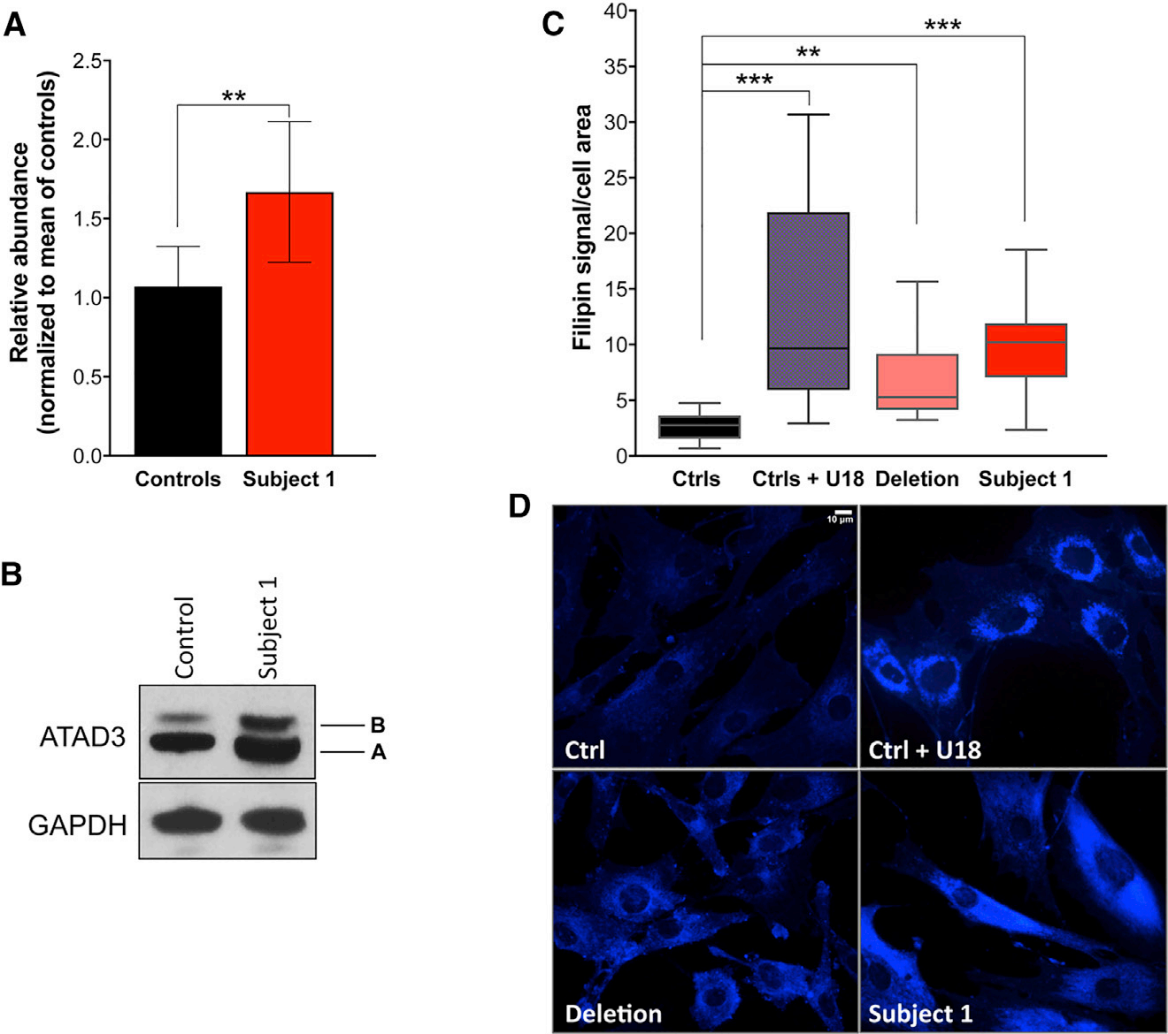
Elevated ATAD3 and Free Cholesterol Levels in Fibroblasts Harboring the *ATAD3* Gene Cluster Duplication (A) Level of ATAD3 in fibroblast of subject 1 compared to control subjects (Fiji ImageJ densitometric analysis). The data are the mean of n = 6 independent experiments using three different control cell lines. Error bars show 1 standard deviation (^∗∗^p < 0.01; Welch’s t test). (B) A representative ATAD3 immunoblot using a pan-specific antibody in fibroblasts. Levels of GAPDH were used as indicators of protein loading. The increased signal of the upper band [B] is consistent with the duplication of *ATAD3B*. ATAD3A isoform 2 and the predicted ATAD3A-C fusion protein are of identical size; hence, the increased signal of the lower band [A] is consistent with the fusion gene being expressed and stable. (C) Chart showing mean filipin signal of cells quantified by ImageJ. Subject 1: fibroblasts of an individual with the *ATAD3* gene cluster duplication; Deletion: fibroblasts of an individual with a biallelic *ATAD3* gene cluster deletion (see Desai et al.^2^ for details); U18: U18666A is an inhibitor of cholesterol trafficking; Filipin is a fluorescent marker, which binds specifically to unesterified cholesterol. Data are the results of 8 independent experiments for subject 1 and control subject(s) and n = 6 for the “deletion.” Error bars show 1 standard deviation (^∗∗∗^p < 0.001; ^∗∗^p ≤ 0.01; one-way ANOVA). (D) Representative images of filipin-stained cells. Scale bar 10 μm.

To experimentally assess the predictions of the *in silico* analyses we amplified a ∼1.8 kb product by reverse transcription PCR on RNA extracted from fibroblasts (subject 4), using a primer pair specific to *ATAD3A* and *ATAD3C*. Sanger sequencing of this product showed a sequence identical to the predicted *ATAD3A-C* transcript (Figure S6). We found that the 5′ region of the *ATAD3A-C* fusion transcript corresponds to that of *ATAD3A*, splicing isoform two. Western blotting showed that fibroblasts (subject 1) harboring the duplication had higher expression of *ATAD3*, compared to controls (Figures 2A and 2B). The upper of the two bands is where ATAD3B migrates and so the increased signal is attributed to the additional copy of *ATAD3B*. As *ATAD3A* is not fully duplicated, the increased signal of the lower band suggests that the *ATAD3A-C* protein product is expressed and stable. ATAD3 is an established mitochondrial protein,^7^ and antibody labeling of ATAD3 in fibroblasts of subject 1 revealed a distribution similar to control cells and to the mitochondrial outer membrane protein TOMM20 (Figure S7). Therefore, both the duplicated *ATAD3B* and the *ATAD3A-C* fusion gene protein product appear to be targeted to the mitochondria.

**Figure 3:**
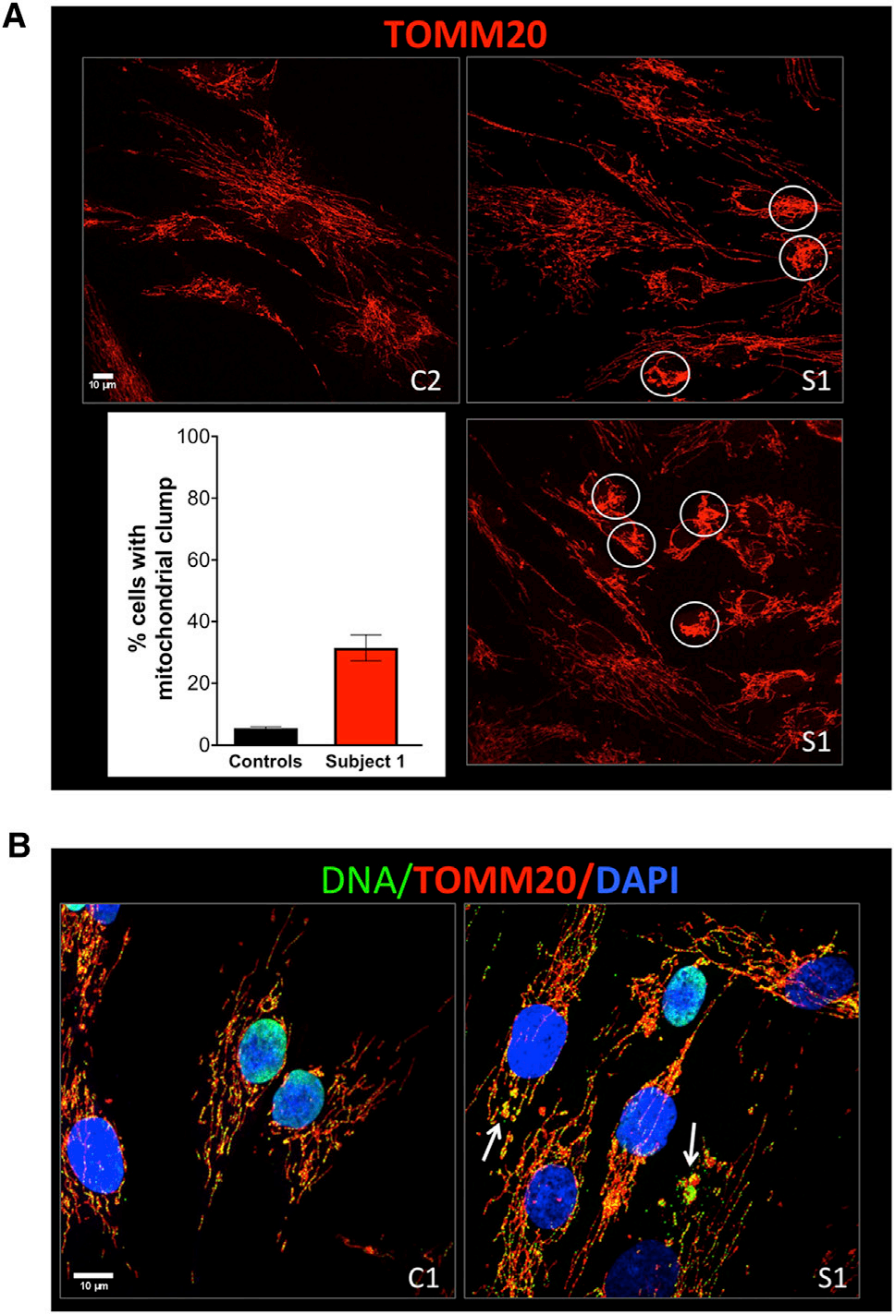
Abnormal Mitochondrial Morphology and mtDNA Organization in Cells with an *ATAD3* Gene Cluster Duplication (A) Confocal images showing the mitochondria of control cell lines (C2) and fibroblasts from subject 1 (S1) labeled with an antibody to the outer mitochondrial membrane protein TOMM20 (red). Proportion of cells with clumped mitochondria for subject 1 versus 2 control subjects (n = 2 independent experiments, ≥50 cells per cell line, per experiment). (B) Fibroblast cells from control subject (C1) and subject 1 (S1) labeled with an antibody against TOMM20 (red), a DNA antibody (green), and DAPI (blue); arrows indicate mtDNA aggregation. Scale bars 10 μm. Error bars show 1 standard deviation.

Variants in *bor*, an *ATAD3A* homolog in *Drosophila melanogaster*, are associated with a reduction in the number of mitochondria and mitochondrial structural abnormalities^1^ and bi-allelic *ATAD3* cluster deletions have been shown to cause mitochondrial structural abnormalities and impaired cholesterol metabolism in human fibroblasts.^2^ Therefore, we assessed mitochondrial morphology and cholesterol levels in our cellular models. In subject 1-derived fibroblasts, free-unesterified cholesterol assessed by filipin staining was significantly higher than control subjects and was similar to cells with pronounced ATAD3 deficiency caused by bi-allelic *ATAD3* cluster deletions^2^ (Figures 2C and 2D). Many fibroblasts (subject 1) showed aggregations of mitochondria, and swollen and rounded organelles (Figure 3A; circled). Nevertheless, cells with an extensive and interconnected mitochondrial network were also apparent (Figure 3A). Immuno-staining for DNA indicated that the swollen mitochondria contained accumulations of mtDNA (Figure 3B; arrows). These features are similar to those associated with *ATAD3* cluster deletions;^2^ we therefore infer that ATAD3A-C is dysfunctional and disrupts mitochondrial morphology and mtDNA organization and causes abnormalities in cellular cholesterol metabolism.

In addition to creating the ATAD3A-C fusion protein, the duplication creates an additional copy of *ATAD3B*. To determine whether this may have contributed to the subjects’ phenotype, exome sequence data from all individuals in the Deciphering Developmental Disorders (DDD) cohort (n = 32,369) were evaluated for the presence of duplications affecting the *ATAD3* cluster. Excluding subject 5, who was identified in this cohort, 61 individuals were identified with likely monoallelic duplications intersecting the *ATAD3* cluster (size range of 67 kb–1.53 Mb), of which 48 affected only the *ATAD3* cluster. All duplications were found to fully encompass *ATAD3B* but did not intersect *ATAD3A.* Duplications were carried either by unaffected parents or probands whose clinical features were inconsistent with a probable metabolic disorder, were above the age of 1 year at their last clinical assessment, and were alive at the point of recruitment. Confirmation testing was not undertaken for the apparently benign duplications, and the precise breakpoints have not been determined. Nevertheless, the presence of multiple *ATAD3B* duplications in this study cohort suggests that the duplication of *ATAD3B* and increased *ATAD3B* gene dosage alone is not causative of this severe phenotype, but rather the NAHR-derived recombinant *ATAD3A-C* gene and novel protein product generated by the *de novo* mutational event.

We have identified two *de novo* duplications within the *ATAD3* cluster in five unrelated individuals whose clinical presentation suggested a metabolic disorder. *ATAD3* gene defects were recently recognized as a cause of human disease,^1^, ^2^, ^3^ accounting for a growing number of phenotypes and cases. Dominant-negative *ATAD3A* missense variants have been reported in individuals affected with hypotonia, optic atrophy, axonal neuropathy, hypertrophic cardiomyopathy, and hereditary spastic paraplegia.^1^^,^^3^ Bi-allelic *ATAD3* cluster deletions result in a more severe phenotype with pontocerebellar hypoplasia^2^^,^^14^^,^^15^ and death in the majority of case subjects within the first week of life similar to bi-allelic *ATAD3A* deletions.^1^ These case subjects with a monoallelic *ATAD3* gene cluster duplication extend the genotype spectrum of *ATAD3*-related disorders.

The phenotype of the neonates with *ATAD3* duplications shows overlap with the previously reported cases associated with pathogenic variation at this locus noting corneal clouding, cardiomyopathy, hypotonia, white matter changes, seizures, fetal akinesia, and contractures. All subjects with duplication died within 6 weeks of life. Although four neonates had low Apgar scores and required intensive clinical management from birth, subject 4 was born prematurely (33+3 weeks), achieved high Apgar scores, had a less severe perinatal course, and presented 3 weeks later with severe lactic acidosis (Table 1 and Supplemental Note). The subjects did not present with obvious signs of mitochondrial distress, and this study highlights the importance of considering mitochondrial genes even in atypical cases, such as these.

**Figure 4:**
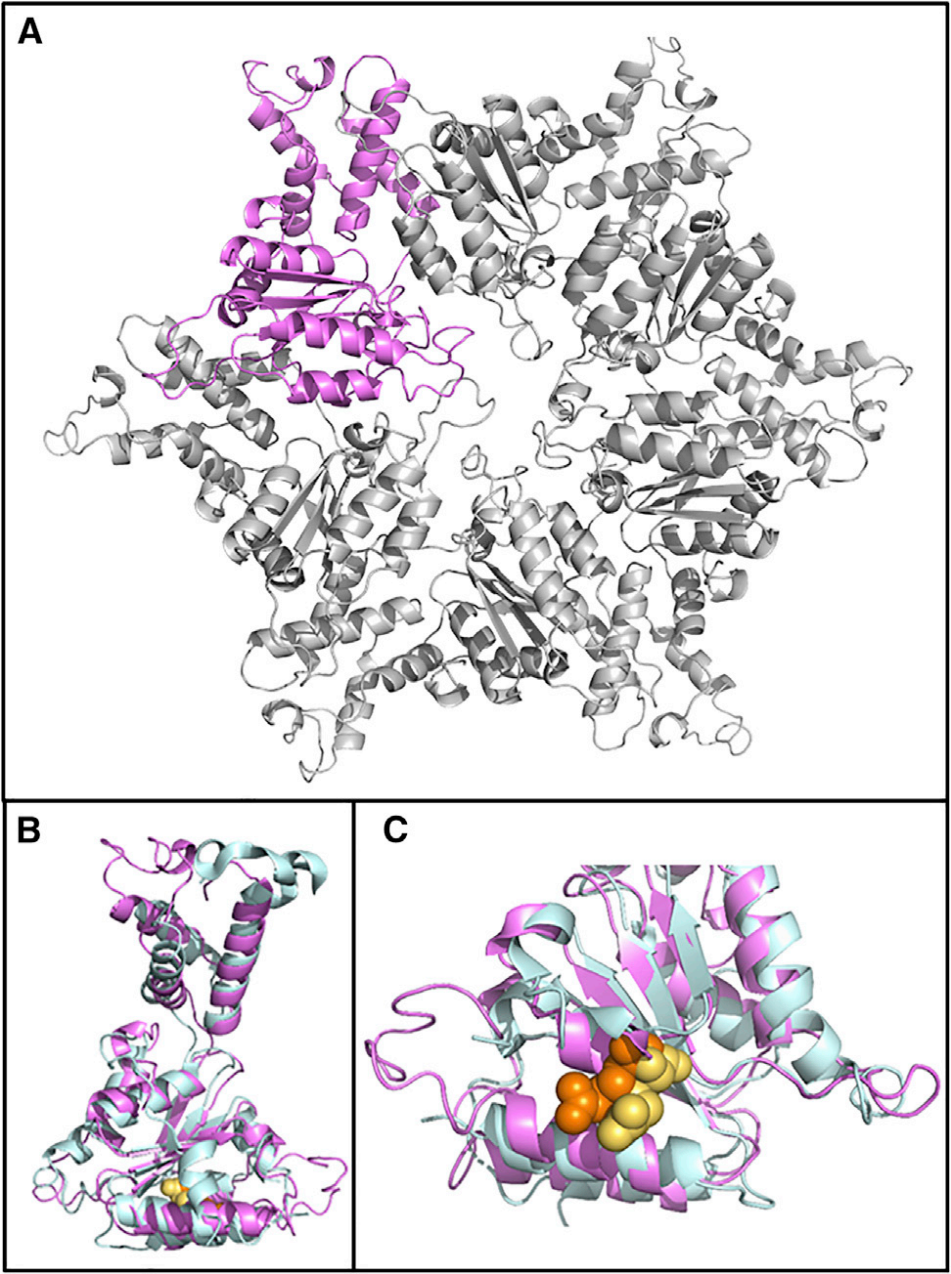
Protein Modeling of ATAD3 Hexamer and 3D Alignment against SPAST ATPase Domain (A) Hexameric structure of ATAD3A ATPase domain (amino acids 348–474), modeled in SwissModel using PDB: 6f0x (*H. sapiens*, TRIP13) as a template. A single monomer is highlighted in violet. (B) Single ATAD3A monomer (violet) aligned to *H. sapiens* SPAST ATPase domain (blue). (C) The ATAD3A arginine finger, Arg466 (yellow) which is changed to a cysteine in the ATAD3A-C fusion gene, is overlaid with the SPAST arginine finger (Arg499; orange).

The *ATAD3A-C* fusion protein is uniquely associated with the severe neonatal phenotype and therefore is likely causal. It is expressed and stable (Figures 2A, 2B, and S6) and has the correct subcellular localization (Figure S7). The fusion protein differs from ATAD3A at 29 amino acid residues within the C-terminal region, including a highly conserved residue within the ATPase domain, p.Arg466Cys (Figure 1D; arrow and Figure 1E). The equivalent residue is conserved in all multimeric AAA-domain containing ATPases and functions as an arginine finger, a *trans*-acting residue that binds to the γ-phosphate of ATP in the neighboring monomer.^16^ Multiple recurrent missense variants have been reported at the equivalent arginine finger residue, Arg499, in SPAST (Figure 4) and cause autosomal-dominant hereditary spastic paraplegia (SPG4 [MIM: 182601]).^17^^,^^18^ These variants have been shown to result in the complete loss of SPAST ATPase activity,^19^ leading to disease through a dominant-negative mechanism. We suggest that the *ATAD3* duplications described here act through the same mechanism: through incorporation of a non-functional monomer derived from the novel fusion protein into more than 90% of ATAD3 hexamers (Figure S5).

Our data suggest that the generation of the fusion protein causes this lethal neurological disorder through disruption of mitochondrial and cholesterol metabolism (Figures 2C, 2D, and 3). This reinforces the links between ATAD3, cholesterol, and mtDNA metabolism. Considering the majority of the cholesterol in mitochondrial membranes co-purifies with mtDNA,^20^ and increasing or decreasing cholesterol availability markedly alters mtDNA organization,^2^ then cholesterol dyshomeostasis evidently disrupts mtDNA metabolism. ATAD3 has links to cholesterol metabolism through partner proteins, TSPO, CYP11A1, and SPTLC.^6^^,^^8^ ATAD3 also co-purifies with the mitochondrial protein synthesis machinery, mtDNA, and mitochondrial cholesterol,^7^^,^^8^^,^^20^ and there is evidence that the mitochondrial nucleoprotein complexes are interlinked.^21^^,^^22^ Hence perturbed cholesterol-containing micro-domains could be the common factor linking all the features associated with ATAD3 deficiency.

Copy number variants (CNVs) pose a practical challenge in genomic analysis, in both their detection and interpretation. Determining how to analyze and interpret rare CNVs which intersect common benign CNVs is not trivial. The high frequency of benign duplications seen in the *ATAD3* region coupled with high sequence homology of the three genes means that pathogenic duplications could potentially be missed. This study highlights the importance of systematic CNV analysis, particularly of genomic intervals prone to instability, where a clinical presentation is consistent with a monogenic disorder.

The high frequency at which this specific *ATAD3* duplication was identified within this cohort suggests that for all clinical suspicions of severe neonatal disorder of unknown origin, negative for known mitochondrial variants and mitochondrial nuclear genome panels, the *ATAD3* locus should be carefully evaluated for single nucleotide, copy-number, and structural variants.

## Declaration of Interests

Baylor College of Medicine (BCM) and Miraca Holdings have formed a joint venture with shared ownership and governance of Baylor Genetics (BG), which performs clinical microarray analysis and clinical exome sequencing. J.R.L. serves on the Scientific Advisory Board of BG. J.R.L. has stock ownership in 23andMe, is a paid consultant for Regeneron Pharmaceuticals, has stock options in Lasergen, and is a co-inventor on multiple United States and European patents related to molecular diagnostics for inherited neuropathies, eye diseases, and bacterial genomic fingerprinting. The other authors declare no competing interests.

## Web Resources

CBS DeepLoc, http://www.cbs.dtu.dk/services/DeepLoc/
DECIPHER, https://decipher.sanger.ac.uk/
EBI KAlign, https://www.ebi.ac.uk/Tools/msa/kalign/
EBI MUSCLE, https://www.ebi.ac.uk/Tools/msa/muscle/
Ensembl Genome Browser, http://www.ensembl.org/index.html
OMIM, https://www.omim.org/
Swiss-Model, https://swissmodel.expasy.org/
Uniprot, https://www.uniprot.org/

## Supplemental Information

Document S1. Figures S1–S8, Supplemental Note, and Supplemental Material and Methods

Document S2. Article plus Supplemental Information
