# Supplementary material for: Recurrent De Novo NAHR Reciprocal Duplications in the ATAD3 Gene Cluster Cause a Neurogenetic Trait with Perturbed Cholesterol and Mitochondrial Metabolism
Source: Am J Hum Genet. 2020 Jan 30;106(2):272–9. doi: 10.1016/j.ajhg.2020.01.007 (PMC7010973; doi:10.1016/j.ajhg.2020.01.007)
Supplement: Document S1. Figures S1–S8, Supplemental Note, and Supplemental Material and Methods [file mmc1.pdf]

The American Journal of Human Genetics, Volume *106*

Supplemental Data

Recurrent *De Novo* NAHR Reciprocal Duplications in the *ATAD3* Gene Cluster Cause a Neurogenetic Trait with Perturbed Cholesterol and Mitochondrial Metabolism

Adam C. Gunning, Klaudia Strucinska, Mikel Muñoz Oreja, Andrew Parrish, Richard Caswell, Karen L. Stals, Romina Durigon, Karina Durlacher-Betzer, Mitchell H. Cunningham, Christopher M. Grochowski, Julia Baptista, Carolyn Tysoe, Emma Baple, Nayana Lahiri, Tessa Homfray, Ingrid Scurr, Catherine Armstrong, John Dean, Uxoa Fernandez Pelayo, Aleck W.E. Jones, Robert W. Taylor, Vinod K. Misra, Wan Hee Yoon, Caroline F. Wright, James R. Lupski, Antonella Spinazzola, Tamar Harel, Ian J. Holt, and Sian Ellard

Supplemental Materials

Supplemental note – Case reports

Figure S1. Exome read depth graph showing the *ATAD3* duplication

Figure S2. ArrayCGH copy-number findings for subject 4

Figure S3. *In silico* splicing prediction of the *ATAD3A-C* fusion gene

Figure S4. Amino acid sequence alignment of ATAD3A and ATAD3A-C

Figure S5. Predicted binomial distribution of ATAD3A and ATAD3A-C in ATAD3 hexameric structures

Figure S6. Nucleotide sequence alignment of a predicted *ATAD3A-C* fusion transcript and RT-PCR DNA product obtained from fibroblasts harboring the duplication

Figure S7. Antibody labelling of ATAD3 in fibroblasts reveals a similar distribution to control cells suggesting the ATAD3A-C fusion protein is targeted to mitochondria

Supplemental Methods

Supplemental References

Supplemental note – Case Reports

Subject 1 was a male child born at term with normal antenatal screening. Fetal akinesia was reported at 38+3 weeks gestation. At birth the child was bradycardic, with Apgars of 3. The child was resuscitated, intubated and transferred to a neonatal unit. He showed severe neonatal encephalopathy with seizures, bilateral corneal opacities, small penis and undescended testes. Renal ultrasound showed pelvicalyceal dilation. Echocardiogram showed poor left ventricular function and thickened right ventricle with abnormal trabeculation of the right ventricular apex. ArrayCGH, Prader-Willi syndrome and spinomuscular atrophy testing were normal. Extensive metabolic investigation showed increased excretion of fumarate, malate, 2-ketoglutarate, 3-methylglutaconate and 3-methylglutarate. Death occurred at three days of age.

Subject 2 was a female child born at 38 weeks with bilateral corneal opacities and bilateral single palmar creases. Antenatally, the neonate showed increased nuchal translucency but no evidence of trisomies 13, 18 or 21. There were poor Apgars at birth and the child was admitted to a neonatal intensive care unit. She developed hypoglycaemia and seizures; EEG analyses showed multifocal cerebral dysfunction; MRI brain showed simplified gyral patterning, temporal cysts and white matter changes. Cardiorespiratory arrest occurred at two weeks. Afterwards, cardiomyopathy was noted, acute renal failure and increasingly oedematous. Death occurred at six weeks of age.

Subject 3 was a male child born at term with normal antenatal screening. Apgars of 1 at birth. The child was grossly hydropic and was resuscitated, intubated ventilated and transferred to a neonatal intensive care unit where abnormal cranial ultrasound with encephalopathy was noted. Death occurred at 5 days of age. Post-mortem studies showed an enlarged dilated heart with endocardial fibrosis and focal myocyte necrosis and widespread hypoxic brain damage.

Subject 4 was a female child born at 33+3 weeks gestation who initially had mild feeding difficulties and hypotonia. She developed severe lactic acidosis at three weeks of life with worsening hypotonia requiring intubation. She was noted to have corneal clouding. She developed a significant pericardial effusion, persistent severe lactic acidosis, and death occurred at six weeks. During that time, a number of studies were done. An EEG showed diffuse cerebral dysfunction. A brain MRI demonstrated diffuse bilateral abnormal subcortical, periventricular, and deep white matter. Brain MR spectroscopy demonstrated decreased N-acetyl aspartate peak, markedly increased lactate peak, and small glutamine-glutamate peak. An echocardiogram showed concentric left ventricular hypertrophy with severely decreased function. Metabolic evaluations and a chromosomal microarray were nondiagnostic, except for urine organic acids, which showed markedly increased lactic acid, moderately increased 2-hydroxybutyric acid, fumaric acid, and 3-hydroxyisobutyric acids. A 202 gene Mitochondrial Genome Plus Mitochondrial Focused Nuclear Gene Panel (GeneDx, Gaithersburg, MD) revealed a heterozygous maternally inherited likely pathogenic variant in *DNM1L* (c.1588C>T; p.(Arg530Ter)). A second *DNM1L* variant was not identified and this maternally-inherited variant was therefore not considered causative. Electron transport chain enzyme studies on skin fibroblasts and very long chain fatty acids with plasmalogens on plasma and skin fibroblasts were normal.

Subject 5 was a male child born at term by Neville Barnes forceps due to a poor cardiotocography. He was hypotonic with bradycardia and had no respiratory effort at birth, but responded to IPPV and chest compression, and was crying with good respiratory effort at 15 minutes. Dense bilateral corneal clouding was noted, and there were mild limb contractures, and glandular hypospadias. He had generalized seizures at 4 days of life, treated with phenobarbitone. Respiratory effort was poor, and he was re-intubated. There may have been antenatal seizures, as daily episodes of fetal hiccoughing were described in the third trimester. An LP showed no evidence of infection, an EEG on

day 6 showed a burst suppression pattern, and a brain MRI demonstrated abnormal white matter and a mild reduction in brain volume. MR spectroscopy showed an increased lactate peak. Multiple renal cysts were noted on ultrasound. An echocardiogram showed a small ventricular septal defect. Although he had no further seizures, he remained hypotonic, with poor respiratory effort and had intermittent metabolic acidosis with raised lactate. At 3 weeks of age, he developed poor perfusion with deteriorating metabolic acidosis and echocardiography revealed an enlarged, dilated heart with wall thickening and a pericardial effusion. Despite inotropic support, diuretics, and respiratory support he did not improve. He died at 4 weeks, 5 days of age. Extensive metabolic investigation was undertaken in life, but was not conclusive. This included urine organic acids which were normal on day 3, but on day 22 there was increased lactate with moderate ketonuria along with a slight increase in malate and fumarate, possibly reflecting impaired mitochondrial function. At post mortem, cardiac muscle histochemistry showed a mosaic pattern of cytochrome c oxidase (COX) deficiency. A generally normal pattern was seen in skeletal muscle although there were some COX-deficient blood vessels. COX (mitochondrial complex IV) enzyme activity was deficient in homogenised heart muscle with sparing of complex II activity.

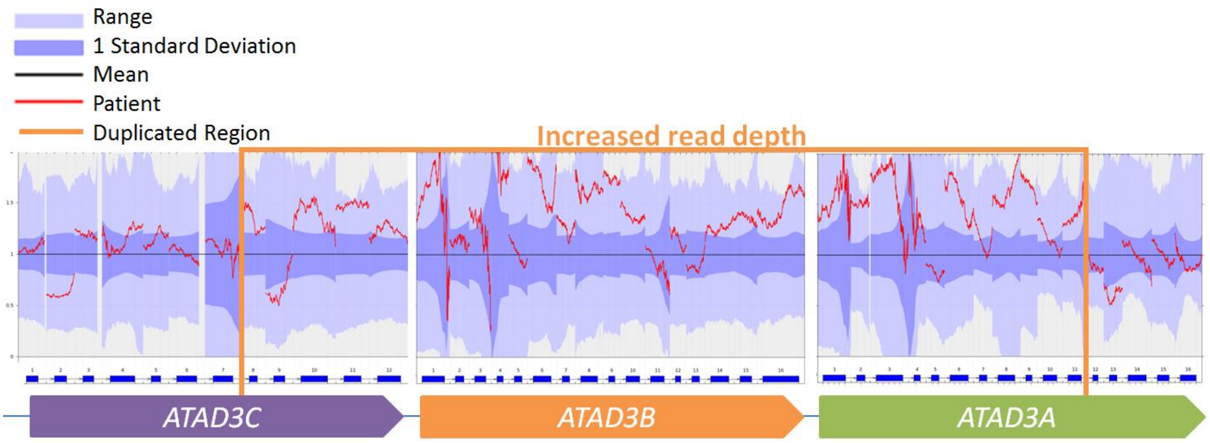

Figure S1. Exome read depth graph showing the *ATAD3* duplication. Normalized read-depth over the ATAD3 cluster for subject 1 (red line), compared to 1,634 samples previously analysed using the same capture library (Mean: Black Line; Range: light blue; 1 Standard Deviation: Dark Blue). The predicted extent of the duplication is shown in orange.

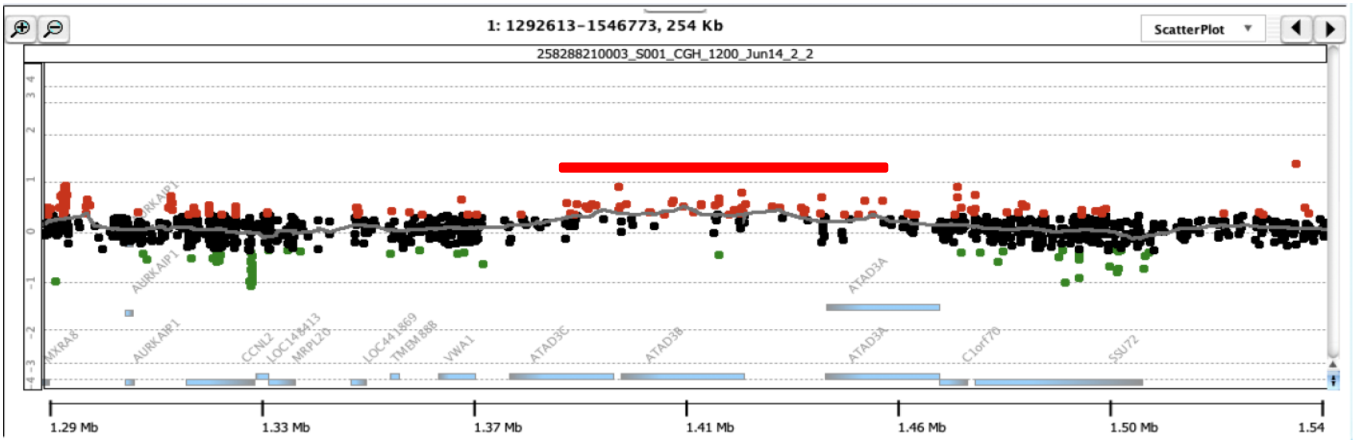

Figure S2. ArrayCGH copy-number findings for subject 4. The predicted duplicated region is represented by a red bar. There remain data-points within the normal range (black) indicating that the duplication is likely heterozygous.

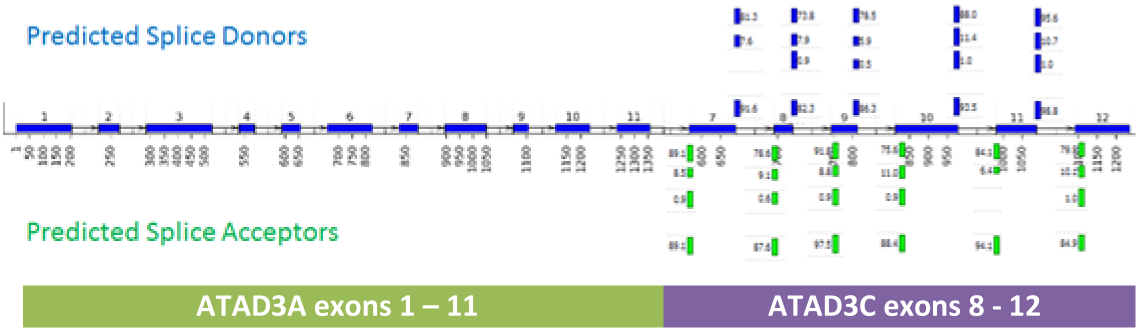

Figure S3. *In silico* splicing prediction of the *ATAD3A-C* fusion gene. Splicing prediction for the *ATAD3A-C* fusion gene, using the (1) SpliceSiteFinder-like, (2) MaxEntScan, (3) Human Splicing Finder algorithms. *ATAD3A* exon 11 and *ATAD3C* exon 7 are homologous. The plot shows that *ATAD3C* retains the splice sites necessary for correct mRNA processing.

ATAD3A

***ATAD3A* exons 1 – 11** | ***ATAD3C* exons 8 - 12**

```
ATAD3A   1   MSWLFGINKGPKGEGAGPPPPLPPAQPGAEGGGDRGLGDRPAPKDKWSNFDPTGLERAAK
             ||||||||||||||||||||||||||||||||||||||||||||||||||||||||||||
Fusion   1   MSWLFGINKGPKGEGAGPPPPLPPAQPGAEGGGDRGLGDRPAPKDKWSNFDPTGLERAAK

ATAD3A   61  AARELEHSRYAKDALNLAQMQEQTLQLEQQSKLKEYEAAVEQLKSEQIRAQAEERRKTLS
             ||||||||||||||||||||||||||||||||||||||||||||||||||||||||||||
Fusion   61  AARELEHSRYAKDALNLAQMQEQTLQLEQQSKLKEYEAAVEQLKSEQIRAQAEERRKTLS

ATAD3A   121 EETRQHQARAQYQDKLARQRYEDQLKQQQLLNEENLRKQEESVQKQEAMRRATVEREMEL
             ||||||||||||||||||||||||||||||||||||||||||||||||||||||||||||
Fusion   121 EETRQHQARAQYQDKLARQRYEDQLKQQQLLNEENLRKQEESVQKQEAMRRATVEREMEL

ATAD3A   181 RHKNEMLRVEAEARARAKAERENADIIREQIRLKAAEHRQTVLESIRTAGTLFGEGFRAF
             ||||||||||||||||||||||||||||||||||||||||||||||||||||||||||||
Fusion   181 RHKNEMLRVEAEARARAKAERENADIIREQIRLKAAEHRQTVLESIRTAGTLFGEGFRAF

ATAD3A   241 VTDWDKVTATVAGLTLLAVGVYSAKNATLVAGRFIEARLGKPSLVRETSRITVLEALRHP
             ||||||||||||||||||||||||||||||||||||||||||||||||||||||||||||
Fusion   241 VTDWDKVTATVAGLTLLAVGVYSAKNATLVAGRFIEARLGKPSLVRETSRITVLEALRHP

ATAD3A   301 IQVSRRLLSRPQDALEGVVLSPSLEARVRDIAIATRNTKKNRSLYRNILMYGPPGTGKTL
             ||||||||||||||||||||||||||||||||||||||||||||||||||||||||||||
Fusion   301 IQVSRRLLSRPQDALEGVVLSPSLEARVRDIAIATRNTKKNRSLYRNILMYGPPGTGKTL

ATAD3A   361 FAKKLALHSGMDYAIMTGGDVAPMGREGVTAMHKLFDWANTSRRGLLLFVDEADAFLRKR
             ||||||||||||||||||||||||||||||||||||||||||||||||||||||||||||
Fusion   361 FAKKLALHSGMDYAIMTGGDVAPMGREGVTAMHKLFDWANTSRRGLLLFVDEADAFLRKR

ATAD3A   421 ATEKISEDLRATLNAFLYRTGQHSNKFMLVLASNQPEQFDWAINDRINEMVHFDLPGQEE
             |||||||||||||||||||||||||||||:|||..|||||||||..|:.|||||||||||
Fusion   421 ATEKISEDLRATLNAFLYRTGQHSNKFMLILASCHPEQFDWAINACIDVMVHFDLPGQEE

ATAD3A   481 RERLVRMYFDKYVLKPATEGKQRLKLAQFDYGRKCSEVARLTEGMSGREIAQLAVSWQAT
             |.||||||.::||||||||||:|||||||||||||.|:||||||||.|:|||||||||||
Fusion   481 RARLVRMYLNEYVLKPATEGKRRLKLAQFDYGRKCLEIARLTEGMSCRKIAQLAVSWQAT

ATAD3A   541 AYASEDGVLTEAMMDTRVQDAVQQHQQKMCWLKAEGPGRGDEPSPS 586
             ||||:||||||||||..|||.||||||.|.|||.|.||..||...|
Fusion   541 AYASKDGVLTEAMMDACVQDFVQQHQQMMRWLKGERPGPEDEQPSS 586
```

Figure S4. Amino acid sequence alignment of ATAD3A and ATAD3A-C. The predicted amino acid sequence of the ATAD3A-C fusion protein (green and purple) aligned against ATAD3A (black). A bar ('|') indicates an identical amino acid, a colon (':') indicates a strongly conservative amino acid change (score > 0.5 in the PAM250 matrix) and a period ('.') indicates a weakly conservative amino acid change (score ≤ 0.5 in the PAM250 matrix). The green residues are derived from the *ATAD3A* gene, while the purple residues are derived from *ATAD3C*. The sequences are of identical length and differ at 29 amino acid positions (highlighted in yellow). Two ATP-binding residues of known function are outlined in red; Asn454 and Arg466. Underlining in the ATAD3A sequence indicates residues of the conserved protein kinase domain [p.Ile348 – p.Asp474; PFam PF00004]. Residue numbering from [Q9NVI7-2 / NM_001170535.2].

Copies of fusion protein in hexamer	Proportion
0	8.8%
1	26.3%
2	32.9%
3	21.9%
4	8.2%
5	1.6%
6	0.1%

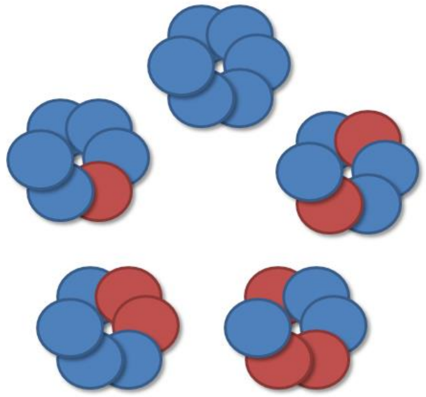

Figure S5. Predicted binomial distribution of ATAD3A and ATAD3A-C in ATAD3 hexameric structures. (Left) The predicted binomial distribution of ATAD3A-C monomers in a homo-hexameric ATAD3 quaternary structure, assuming a single copy of *ATAD3A-C* and two copies of *ATAD3A*. **(Right)** A cartoon showing the incorporation of non-functional ATAD3A-C monomers in the ATAD3 hexamer.

```
predicted      atgtcgtggctcttcggcattaacaagggccccaagggtgaaggcgcggggccgccgccg      60
RT-PCR         atGTCGTGGCTCTTCGGCATTAACAAGGGCCCCAAGGGTGAAGGCGCGGGGCCGCCGCCG      60
               ************************************************************

predicted      cctttgccgcccgcgcagcccggggccgagggcggcggggaccgcgggttgggagaccgg      120
RT-PCR         CCTTTGCCGCCCGCGCAGCCCGGGGCCGAGGGCGGCGGGGACCGCGGGTTGGGAGACCGG      120
               ************************************************************

predicted      ccggcgcccaaggacaaatggagcaacttcgacCCCACCGGCCTGGAGCGCGCCgccaag      180
RT-PCR         CCGGCGCCCAAGGACAAATGGAGCAACTTCGACCCCACCGGCCTGGAGCGCGCCGCCAAG      180
               ************************************************************

predicted      gcggcgcgcgagctggagcactcgcgttatgccaaggacgccctgaatctggcacagatg      240
RT-PCR         GCGGCGCGCGAGCTGGAGCACTCGCGTTATGCCAAGGACGCCCTGAATCTGGCACAGATG      240
               ************************************************************

predicted      caggagcagacgctgcagttggagcaacagtccaagctcaaagagtatgaggccgccgtg      300
RT-PCR         CAGGAGCAGACGCTGCAGTTGGAGCAACAGTCCAAGCTCAAAGAGTATGAGGCCGCCGTG      300
               ************************************************************

predicted      gagcagctcaagagcgagcagatccgggcgcaggctgaggagaggaggaagaccctgagc      360
RT-PCR         GAGCAGCTCAAGAGCGAGCAGATCCGGGCGCAGGCTGAGGAGAGGAGGAAGACCCTGAGC      360
               ************************************************************

predicted      gaggagacccggcagcaccAGGCCAGGGCCCAGTATCAAGacaagctggcccggcagcgc      420
RT-PCR         GAGGAGACCCGGCAGCACCAGGCCAGGGCCCAGTATCAAGACAAGCTGGCCCGGCAGCGC      420
               ************************************************************

predicted      tacgaggaccaactgaagcagcagcaacttctcaatgaggagaatttacggaagcaggag      480
RT-PCR         TACGAGGACCAACTGAAGCAGCAGCAACTTCTCAATGAGGAGAATTTACGGAAGCAGGAG      480
               ************************************************************

predicted      gagtccgtgcagaagcaggaagccatgcggcgagccaccgtggagcggGAGATGGAGCTG      540
RT-PCR         GAGTCCGTGCAGAAGCAGgaagccatgcggcgagccaccgtggagcgggagatggagctg      540
               ************************************************************

predicted      CGGCACAAGaatgagatgctgcgagtggaggccgaggcccgggcgcgcgccaaggccgag      600
RT-PCR         cggcacaagaatgagatgctgcgagtggaggccgaggcccgggcgcgcgccaaggccgag      600
               ************************************************************

predicted      cgggagaatgcagacatcatccgcgagcagatccgcctgaaggcggccgagcaccgtcag      660
RT-PCR         cgggagaatgcagacatcatccgcgagcagatccgcctgaaggcggccgagcaccgtcag      660
               ************************************************************

predicted      accgtcttggagtccatcaggacggctggcaccttgtttggggaaggattccgtgccttt      720
RT-PCR         accgtcttggagtccatcaggacggctggcaccttgtttggggaaggattccgtgccttt      720
               ************************************************************

predicted      gtgacagactgggacaaagtgacagccacggtggctgggctgacgctgctggctgttggG      780
RT-PCR         gtgacagactgggacaaagtgacagccacggtggctgggctgacgctgctggctgttggg      780
               ************************************************************

predicted      GTCTACTCAGCCAAGAATGCcacgcttgtcgccggccgcttcatcgaggctcggctgggg      840
RT-PCR         gtctactcagccaagaatgccacgcttgtcgccggccgcttcatcgaggctcggctgggg      840
               ************************************************************

predicted      aagccgtccctagtgagggagacgtcccgcatcacggtgcttgaggcgctgcggcacccc      900
RT-PCR         aagccgtccctagtgagggagacgtcccgcatcacggtgcttgaggcgctgcggcacccc      900
               ************************************************************

predicted      atccaggtcagccggcggctcctcagtcgaccccaggacgcgctggagggtgttgtgctc      960
RT-PCR         atccaggtcagccggcggctcctcagtcgaccccaggacgcgctggagggtgttgtgctc      960
               ************************************************************

predicted      agtcccagcctggaagcacgggtgcgcgacatcgccatagcaacaaggaacaccaagaag      1020
RT-PCR         agtcccagcctggaagcacgggtgcgcgacatcgccatagcaacaaggaacaccaagaag      1020
               ************************************************************

predicted      aaccgcagcctgtacaggaacatcctgatgtacgggccaccaGGCaccgggAAGacgctg      1080
RT-PCR         aaccgcagcctgtacaggaacatcctgatgtacgggccaccaggcaccgggaagacgctg      1080
               ************************************************************
```

```
predicted      tttgccaagaaactcgccctgcactcaggcatggactacgccatcatgacaggcggggac      1140
RT-PCR         tttgccaagaaactcgccctgcactcaggcatggactacgccatcatgacaggcggggac      1140
               ************************************************************

predicted      gtggcccccatggggcgggaaggcgtgaccgccatgcacAAGCTCTTTGACTGGGCCAAT      1200
RT-PCR         gtggcccccatggggcgggaaggcgtgaccgccatgcacaagctctttgactgggccaat      1200
               ************************************************************

predicted      accagccggcgcggcCTCCTGCTCTTTGTGGATGAAGCGGACGCCTTCCTTCGGAAGCGA      1260
RT-PCR         accagccggcgcggcctcctgctctttgtggatgaagcggacgccttccttcggaagcga      1260
               ************************************************************

predicted      GCCACTgagaagataagcgaggacctcagggccacactgaacgccttcctgtaccgcacg      1320
RT-PCR         gccactgagaagataagcgaggacctcagggccacactgaacgccTTCCTGTACCGCACG      1320
               ************************************************************

predicted      ggccagcacagcaacaaattcatgctgatcctggccagctgccaccccgagcagttcgac      1380
RT-PCR         GGCCAGCACAGCAACAAATTCATGCTGATCCTGGCCAGCTGCCACCCCGAGCAGTTCGAC      1380
               ************************************************************

predicted      tgggccatcaatgcctgcatcgacgtgatggtccacttcgacctgccagggcaggaggag      1440
RT-PCR         TGGGCCATCAATGCCTGCATCGACGTGATGGTCCACTTCGACCTGCCAGGGCAGGAGGAG      1440
               ************************************************************

predicted      cgggcgcgcctggtgagaatgtatcttaacgagtatgttcttaagccggccacagaagga      1500
RT-PCR         CGGGCGCGCCTGGTGAGAATGTATCTTAACGAGTATGTTCTTAAGCCGGCCACAGAAGGA      1500
               ************************************************************

predicted      aagcggcgtctgaagctggcccagtttgactacgggaggaagtgcttagagatcgctcgg      1560
RT-PCR         AAGCGGCGTCTGAAGCTGGCCCAGTTTGACTACGGGAGGAAGTGCTTAGAGATCGCTCGG      1560
               ************************************************************

predicted      ctgacagagggcatgtcatGCCGGAAGATCGCACAGCTGGccgtgtcctggcaggccacg      1620
RT-PCR         CTGACAGAGGGCATGTCATGCCGGAAGATCGCACAGCTGGCCGTGTCCTGGCAGGCCACG      1620
               ************************************************************

predicted      gcgtatgcctccaaggacggggtcctgaccgaggccatgatggacgcctgcgtgcaagac      1680
RT-PCR         GCGTATGCCTCCAAGGACGGGGTCCTGACCGAGGCCATGATGGACGCCTGCGTGCAAGAC      1680
               ************************************************************

predicted      tttgtccagcagcaccagcagatgatgcgctggctgaaggggga gaggcctgggcccgag      1740
RT-PCR         TTTGTCCAGCAGCACCAGCAGATGATGCGCTggctgaagggggagaggcctgggcccgag      1740
               ************************************************************

predicted      gacgagcaaccctcatcctga     1761
RT-PCR         gacgagcaaccctcatcctga     1761
               *********************
```

Figure S6. Nucleotide sequence alignment of a predicted ATAD3A-C fusion transcript and RT-PCR DNA product obtained from fibroblasts harboring the duplication. The predicted nucleotide sequence of the *ATAD3A-C* fusion transcript aligned with that for a reverse transcription PCR (RT-PCR) product obtained from fibroblasts harboring the duplication (subject 4). The sequence obtained matches the predicted fusion transcript exactly.

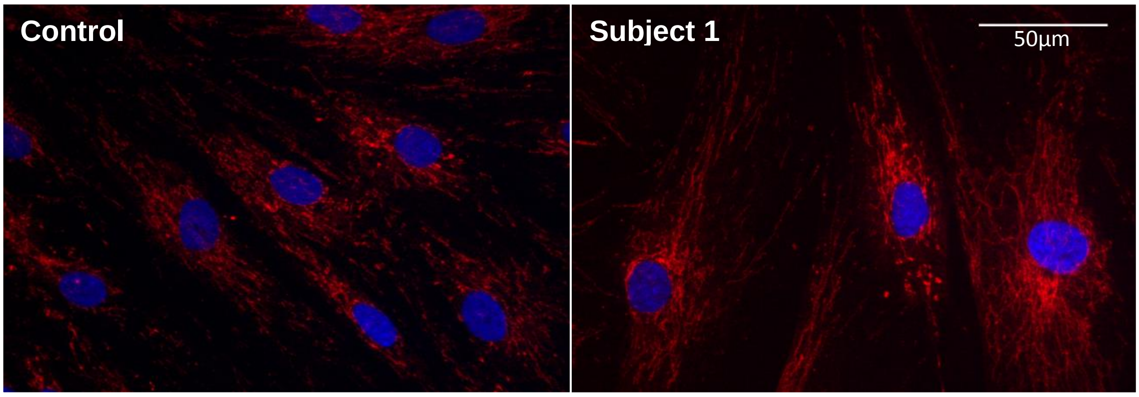

Figure S7. Antibody labelling of ATAD3 in fibroblasts reveals a similar distribution to control cells suggesting the ATAD3A-C fusion protein is targeted to mitochondria. Fibroblasts of control cells and subject 1 were labelled with anti-ATAD3 antibody (red) and DAPI (blue). Note the similar distribution to TOMM20 labeling [***Figure 3A***]. Co-staining cells with anti-ATAD3 and anti-TOMM20 confirmed the ATAD3 signal was restricted to the mitochondria (data not shown).

Supplemental Methods

Subjects

Cases 1-4 were referred for diagnostic trio exome sequencing due to a severe congenital developmental disorder. Case 5 was found through a manual analysis of the *ATAD3* cluster in the Deciphering Developmental Disorders (DDD) study cohort (4) for all participants with an age of death under one year (n=66). Informed consent for testing and publication was obtained from all participants.

Samples and genomic data

Sample collection, DNA extraction, arrayCGH, exome library preparation, trio exome sequencing, variant calling and annotation were performed as described (5). DNA was extracted from cultured skin fibroblasts from subjects one and four. Exome read-depth and arrayCGH data were assessed manually.

Confirmation of results by PCR and Sanger sequencing

Primers were designed using Primer3 (National Human Genome Research Institute, USA). For case four, a forward primer specific to *ATAD3A* intron 9 (Chr1(GRCh38):g.1523723-1523743) and a reverse primer specific to *ATAD3C* intron 8 (Chr1(GRCh38):g.1457228-1457250) were designed. For the other cases, the forward primer was located in *ATAD3A* exon 10 (NC_000001.11(GRCh38):g.1523875-1523893) and the reverse in *ATAD3C* intron 7 (NC_000001.11(GRCh38):g.1456957-1456977). Products were visualized by electrophoresis on a 3% agarose gel and sequenced bi-directionally.

Protein modelling

The crystal structure of the ATAD3 ATPase domain has not been solved; the ATPase domains of ATAD3A [Ensembl: ENST00000378756.8; Uniprot:Q9NVI7-2] and the predicted fusion protein [residues 1-405 of Q9NVI7-2, residues 231-411 of Q5T2N8-1, “ATAD3A-C”] were modelled using SWISS-MODEL (6). The highest scoring structure [PDB: 6f0x; H. sapiens TRIP13] was used as a template for modelling both structures. The models were visualized in PyMol.

In silico analyses

In silico splicing analysis was performed on the splice junctions of ATAD3C exons 8-12, using the SpliceSiteFinder-like (1), MaxEntScan (2) and HSF (3) algorithms. The amino acid sequences of ATAD3A and ATAD3A-C were aligned using MUSCLE (7). The sequences were provided to DeepLoc (8) for analysis of the predicted subcellular localization. The genomic nucleotide sequences surrounding *ATAD3A* and *ATAD3C* were obtained from ENSEMBL and aligned using KAlign (9) to identify regions of homology.

Cell culture

Primary skin fibroblast cultures were obtained from subjects one and four, and healthy control individuals. Samples were confirmed free of mycoplasma based on the LookOut Mycoplasma PCR Detection Kit (Sigma). They were cultured in Dulbecco’s Modified Eagle’s Medium (DMEM, LifeTechnologies) supplemented with 10% fetal bovine serum (FBS, Hyclone/Sigma), 1% penicillin and streptomycin (PS, Life Technologies) at 37°C in a 5% CO_2 atmosphere.

Reverse transcription, and polymerase chain reaction

Total RNA was purified from fibroblasts obtained from subject 4 using TRIzol reagent (Life Technologies). cDNA was generated from 1 μg of total RNA using the SuperScript IV First-Strand Synthesis System (Life technologies). The ATAD3A-C fusion gene was amplified using a forward primer specific to ATAD3A (NC_000001.11(GRCh38):g.1512269-1512287) and a reverse primer specific to ATAD3C (NC_000001.11(GRCh38):g.1468505-1468530).

Western blot analysis, immunofluorescence and cell imaging

Protein fractionation, transfer and immuno-detection were performed as described (10). Cells were lysed on ice in phosphate-buffered saline (PBS), *n*-dodecyl-D-maltoside (DDM), 1X protease inhibitor cocktail (Roche), and phosphatase inhibitor cocktail (Abcam) and 50 Units Benzonase (Millipore). Protein concentration was determined by DC protein assay kit (Biorad). Protein samples were prepared in 1× Laemmli loading buffer, heated at 42°C for 15 minutes and resolved on SDS-PAGE gels (Novex, Thermofisher Scientific). After electrophoresis, proteins were transferred to polyvinylidene fluoride membranes (PVDF, Millipore). Membranes were blocked with 5% non-fat dry milk in PBS with 0.1% (v/v) Tween-20 (PBST) and incubated overnight at 4°C with the primary antibodies: anti-GAPDH (1:20 000, Abcam), anti-ATAD3 (1:50 000, gift from John Walker and Jiuya He).

Fibroblasts grown on coverslips were fixed with 4% paraformaldehyde in PBS for 15 minutes at 37°C. Cells were then washed three times for 5 minutes each with PBS before being permeabilized with 0.3% Triton X-100 in PBS containing 5% fetal bovine serum (PBSS) for 5 minutes at room temperature. After permeabilization, samples were washed and blocked with PBSS for 1 hour at room temperature and later incubated with the indicated primary antibodies: anti-DNA (1:200-250, Progen), anti-TOMM20 (abcam 1:100-500), anti-ATAD3A (1:100, Novusbio) at 4°C overnight. Following washes, cells were incubated for 1 hour at room temperature with secondary antibody, after which coverslips were mounted on glass slides over ProLong Gold Antifade Reagent. Unesterified cholesterol in fibroblasts was stained with filipin, using a cholesterol assay kit (Abcam), detected by wide-field fluorescence microscopy and quantified using ImageJ. Images of filipin stained cells were acquired with a Nikon eclipse 80i epifluorescence microscope, using the NIS elements software. Filipin signals (pixels/unit area) were quantified in Image J, using the Huang algorithm to define the area of the cells.

Supplemental References

1. Shapiro MB, Senapathy P. RNA splice junctions of different classes of eukaryotes: sequence statistics and functional implications in gene expression. Nucleic Acids Res. 1987 Sep 11;15(17):7155–7174.
2. Yeo G, Burge CB. Maximum entropy modeling of short sequence motifs with applications to RNA splicing signals. J Comput Biol. 2004;11(2-3):377–394.
3. Desmet F-O, Hamroun D, Lalande M, Collod-Béroud G, Claustres M, Béroud C. Human Splicing Finder: an online bioinformatics tool to predict splicing signals. Nucleic Acids Res. 2009 May;37(9):e67.
4. Wright CF, Fitzgerald TW, Jones WD, Clayton S, McRae JF, van Kogelenberg M, et al. Genetic diagnosis of developmental disorders in the DDD study: a scalable analysis of genome-wide research data. Lancet. 2015 Apr 4;385(9975):1305–1314.
5. Stals KL, Wakeling M, Baptista J, Caswell R, Parrish A, Rankin J, et al. Diagnosis of lethal or prenatal-onset autosomal recessive disorders by parental exome sequencing. Prenat Diagn. 2018;38(1):33–43.
6. Guex N, Peitsch MC. SWISS-MODEL and the Swiss-PdbViewer: an environment for comparative protein modeling. Electrophoresis. 1997 Dec;18(15):2714–2723.
7. Edgar RC. MUSCLE: multiple sequence alignment with high accuracy and high throughput. Nucleic Acids Res. 2004 Mar 19;32(5):1792–1797.
8. Almagro Armenteros JJ, Sønderby CK, Sønderby SK, Nielsen H, Winther O. DeepLoc: prediction of protein subcellular localization using deep learning. Bioinformatics. 2017 Nov 1;33(21):3387–3395.
9. Lassmann T, Frings O, Sonnhammer ELL. Kalign2: high-performance multiple alignment of protein and nucleotide sequences allowing external features. Nucleic Acids Res. 2009 Feb;37(3):858–865.
10. Dalla Rosa I, Durigon R, Pearce SF, Rorbach J, Hirst EMA, Vidoni S, et al. MPV17L2 is required for ribosome assembly in mitochondria. Nucleic Acids Res. 2014 Jul;42(13):8500–8515.
