# Supplementary material for: Recurrent De Novo NAHR Reciprocal Duplications in the ATAD3 Gene Cluster Cause a Neurogenetic Trait with Perturbed Cholesterol and Mitochondrial Metabolism
Source: Am J Hum Genet. 2020 Jan 30;106(2):272–9. doi: 10.1016/j.ajhg.2020.01.007 (PMC7010973; doi:10.1016/j.ajhg.2020.01.007)
Supplement: Document S2. Article plus Supplemental Information [file mmc2.pdf]

REPORT

Recurrent *De Novo* NAHR Reciprocal Duplications in the *ATAD3* Gene Cluster Cause a Neurogenetic Trait with Perturbed Cholesterol and Mitochondrial Metabolism

Adam C. Gunning,[1,2] Klaudia Strucinska,[3] Mikel Muñoz Oreja,[4] Andrew Parrish,[1] Richard Caswell,[2] Karen L. Stals,[1] Romina Durigon,[5] Karina Durlacher-Betzer,[6] Mitchell H. Cunningham,[7] Christopher M. Grochowski,[8] Julia Baptista,[1,2] Carolyn Tysoe,[1] Emma Baple,[1,2] Nayana Lahiri,[9] Tessa Homfray,[9,10] Ingrid Scurr,[11] Catherine Armstrong,[12] John Dean,[13] Uxoa Fernandez Pelayo,[4] Aleck W.E. Jones,[5] Robert W. Taylor,[14] Vinod K. Misra,[7] Wan Hee Yoon,[3] Caroline F. Wright,[2] James R. Lupski,[8,15,16,17] Antonella Spinazzola,[5,18] Tamar Harel,[6] Ian J. Holt,[4,5,19,20] and Sian Ellard[1,2,*]

Recent studies have identified both recessive and dominant forms of mitochondrial disease that result from *ATAD3A* variants. The recessive form includes subjects with biallelic deletions mediated by non-allelic homologous recombination. We report five unrelated neonates with a lethal metabolic disorder characterized by cardiomyopathy, corneal opacities, encephalopathy, hypotonia, and seizures in whom a monoallelic reciprocal duplication at the *ATAD3* locus was identified. Analysis of the breakpoint junction fragment indicated that these 67 kb heterozygous duplications were likely mediated by non-allelic homologous recombination at regions of high sequence identity in *ATAD3A* exon 11 and *ATAD3C* exon 7. At the recombinant junction, the duplication allele produces a fusion gene derived from *ATAD3A* and *ATAD3C*, the protein product of which lacks key functional residues. Analysis of fibroblasts derived from two affected individuals shows that the fusion gene product is expressed and stable. These cells display perturbed cholesterol and mitochondrial DNA organization similar to that observed for individuals with severe ATAD3A deficiency. We hypothesize that the fusion protein acts through a dominant-negative mechanism to cause this fatal mitochondrial disorder. Our data delineate a molecular diagnosis for this disorder, extend the clinical spectrum associated with structural variation at the *ATAD3* locus, and identify a third mutational mechanism for *ATAD3* gene cluster variants. These results further affirm structural variant mutagenesis mechanisms in sporadic disease traits, emphasize the importance of copy number analysis in molecular genomic diagnosis, and highlight some of the challenges of detecting and interpreting clinically relevant rare gene rearrangements from next-generation sequencing data.

Since its initial association with a neurological disorder,[1] it has become apparent that disruption of the *ATAD3* cluster, and more specifically *ATAD3A* (MIM: 612316), is a significant cause of pediatric disease. Variants at this locus are associated with a wide phenotypic spectrum, including pontocerebellar hypoplasia,[2] hereditary spastic paraplegia,[3] and a syndromic neurological disorder characterized by peripheral neuropathy, hypotonia, cardiomyopathy, optic atrophy, cerebellar atrophy, and seizures:[1] Harel-Yoon syndrome (HAYOS [MIM: 617183]). The different phenotypes can be attributed to a spectrum of disease-causing variants that includes bi-allelic hypomorphic variants, bi-allelic deletions, and monoallelic dominant-negative missense variants. Here, we report two *de novo* intergenic duplications in the *ATAD3* cluster identified in five unrelated neonates with shared phenotypes including corneal clouding, cardiomyopathy, hypotonia, and white matter changes, thus expanding the genotype spectrum of *ATAD3*-related disorders.

The *ATAD3* cluster is composed of three paralogs with extensive sequence homology, formed through tandem segmental duplication: *ATAD3A*, *ATAD3B* (MIM: 612317), and *ATAD3C* (MIM: 617227). *ATAD3A* and *ATAD3B* are protein-coding genes of near identical sequence, differing primarily due to a stop-loss mutation in *ATAD3B* that extends the protein by 62 amino acids; *ATAD3C* is not known to be expressed. ATAD3A is a transmembrane ATPase, which is predicted to form hexamers,[4]

[1]Exeter Genomics Laboratory, Royal Devon and Exeter NHS Foundation Trust, Exeter EX2 5DW, UK; [2]Institute of Biomedical and Clinical Science, College of Medicine and Health, University of Exeter, Exeter EX2 5DW, UK; [3]Aging and Metabolism Research Program, Oklahoma Medical Research Foundation, Oklahoma City, OK 73104, USA; [4]Biodonostia Health Research Institute, 20014 San Sebastián, Spain; [5]Department of Clinical and Movement Neurosciences, UCL Queen Square Institute of Neurology, Royal Free Campus, London NW3 2PF, UK; [6]Department of Genetic and Metabolic Diseases, Hadassah-Hebrew University Medical Center, Jerusalem 91120, Israel; [7]Department of Pediatrics, Division of Genetic, Genomic, and Metabolic Disorders, Wayne State University School of Medicine, Children's Hospital of Michigan, Detroit, MI 48201, USA; [8]Department of Molecular and Human Genetics, Baylor College of Medicine, Houston, TX 77030, USA; [9]South West Thames Regional Genetics Service, St George's University Hospitals NHS Foundation Trust, London SW17 0QT, UK; [10]St George's University of London, London SW17 0RE, UK; [11]Department of Clinical Genetics, University Hospitals Bristol NHS Foundation Trust, Bristol BS2 8EG, UK; [12]Department of Paediatric Cardiology, University Hospitals Bristol NHS Foundation Trust, Bristol BS2 8BJ, UK; [13]Clinical Genetics Service, NHS Grampian, Aberdeen Royal Infirmary, Aberdeen AB25 2ZA, UK; [14]Wellcome Centre for Mitochondrial Research, Translational and Clinical Research Institute, Faculty of Medical Sciences, Newcastle University, Newcastle upon Tyne NE2 4HH, UK; [15]Department of Pediatrics, Baylor College of Medicine, Houston, TX 77030, USA; [16]Human Genome Sequencing Center, Baylor College of Medicine, Houston, TX 77030, USA; [17]Texas Children's Hospital, Houston, TX 77030, USA; [18]MRC Centre for Neuromuscular Diseases, UCL Queen Square Institute of Neurology and National Hospital for Neurology and Neurosurgery, Queen Square, London WC1N 3BG, UK; [19]IKERBASQUE, Basque Foundation for Science, 48013 Bilbao, Spain; [20]CIBERNED (Center for Networked Biomedical Research on Neurodegenerative Diseases, Ministry of Economy and Competitiveness, Institute Carlos III), 28031 Madrid, Spain
*Correspondence: sian.ellard@nhs.net
https://doi.org/10.1016/j.ajhg.2020.01.007.
© 2020 The Authors. This is an open access article under the CC BY license (http://creativecommons.org/licenses/by/4.0/).

Table 1. Clinical Features of Individuals with Duplication in *ATAD3* Gene Cluster

	Subject 1	Subject 2	Subject 3	Subject 4	Subject 5
Sex	male	female	male	female	male
Gestation	term	38 weeks	term	33+3 weeks	term
Apgars at birth	3	poor	1	5,8,9	1,0
Chronological age at death	3 days	6 weeks	5 days	6 weeks	4 weeks
Cardiomyopathy	HCM	DCM	DCM; cardiomegaly	HCM; cardiomegaly	HCM; cardiomegaly
Congenital cataracts	✓	ND	ND	ND	ND
Corneal opacity	✓	✓	✓	✓	✓
Postnatal hypotonia	✓	✓	✓	✓	✓
Abnormality of the external genitalia	cryptorchidism and micropenis	ND	ND	ND	hypospadias
Seizures	✓	diffuse abnormalities on EEG	ND	diffuse abnormalities on EEG	✓
Encephalopathy	✓	ND	✓	ND	ND
Brain findings	ND	white matter changes; simplified gyral patterning; cerebellar atrophy (MRI)	widespread hypoxic brain damage (post-mortem)	diffuse bilateral abnormal subcortical, periventricular, and deep white matter; abnormal MR spectroscopy	white matter changes, generalized reduction of brain volume (MRI); abnormal MR spectroscopy (lactate peak) on day 9
Contractures/ fetal akinesia	fetal akinesia	ND	contractures	ND	contractures
Edema/fetal hydrops	ND	fetal hydrops; edema	fetal hydrops	ND	ND
Metabolic investigations	increased excretion of fumarate, malate, 2-ketoglutarate, 3-methylglutaconate, and 3-methylglutarate	lactic acidosis	ND	lactic acidosis; increased excretion of 2OH butyrate, fumarate, and 3OH isobutyrate	lactic acidosis, increased excretion of fumarate, malate on day 22
Prior genetic investigations	ArrayCGH; Prader-Willi; SMA	prenatal aneuploidy	ND	arrayCGH; 202 gene mitochondrial panel	arrayCGH, 27 gene glycogen storage disease panel

HCM, hypertrophic cardiomyopathy; DCM, dilated cardiomyopathy; SMA, spinal muscular atrophy; ND, not detected.

a fraction of which is found at contact sites between the inner and outer mitochondrial membranes[5] in complex with TSPO, CYP11A1, and OPA1.[6] ATAD3 has also been shown to interact with mitochondrial nucleoprotein complexes and to play roles in mtDNA organization and replication.[2,7,8] More recently it has been shown to interact with Drp1/DNM1L to support Drp1-induced mitochondrial division,[9] a process that drives mtDNA segregation.[10,11] Concordantly, ATAD3 dysfunction and deficiency have a wide range of effects on mitochondrial structure and function, characterized by disturbed mitochondrial morphology and fission dynamics,[3,6] loss of cristae,[12] perturbed mtDNA and cholesterol metabolism, impaired mitochondrial steroidogenesis,[2,13] and decreased levels of some mitochondrial oxidative phosphorylation (OXPHOS) components.[12] It is not clear whether the disruption to the inner mitochondrial membrane, mtDNA, and OXPHOS complexes are due directly to the absence of ATAD3[4,12] or whether they are consequences of changes to membrane architecture resulting from an altered cholesterol content[2,13] or a combination of the two.

We report *de novo ATAD3* duplications identified in five unrelated neonates through exome sequencing. Clinical exome sequencing failed to identify any alternative molecular diagnosis potentially causative of the phenotype, which is characterized by seizures (four of the five neonates) and fetal akinesia and contractures (in three case subjects). A clinical summary is shown in Table 1 and clinical case reports are detailed in the Supplemental Note. Informed consent was obtained and all processes adhered to local and national ethical standards. The duplication in the *ATAD3* cluster was also detected by arrayCGH for those subjects studied (subjects four and five). The duplication is predicted to be the product of

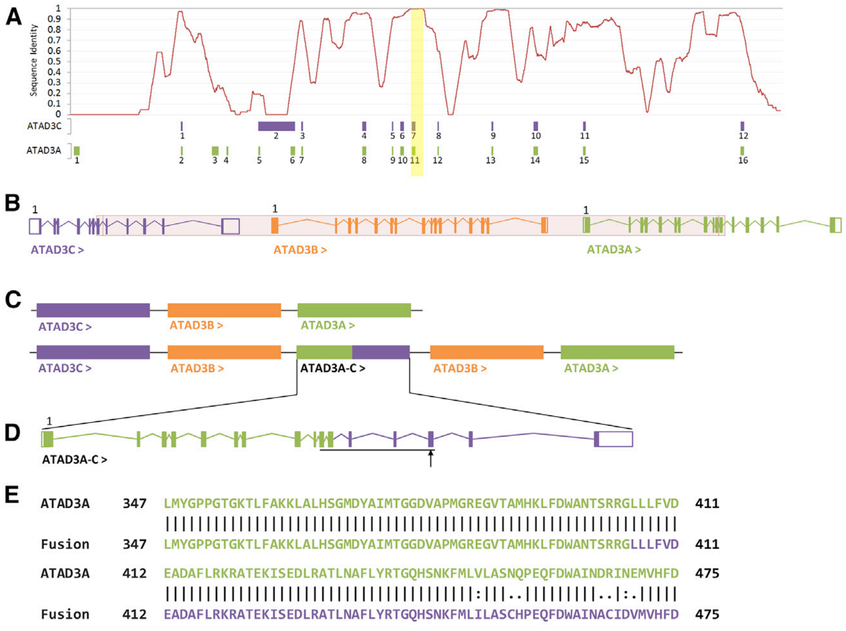

Figure 1. NAHR between *ATAD3C* Exon 8 and *ATAD3A* Exon 11 Produces a Fusion Gene, with Variants at Key Functional Residues within the ATPase Domain

Gene intron-exon structures are shown in cartoon format; open boxes indicate UTRs while closed boxes indicate coding regions. Arrows following the gene name indicate reading direction, and the first exon is labeled. Genes are shown in their relative position on chromosome 1 in a 5′ to 3′ direction from left to right.

(A) Nucleotide sequence identity between *ATAD3A* (chr1:1512151–1534687:1) and *ATAD3C* (chr1:1449689–1470158:1) in a sliding 500 bp window. *ATAD3A* and *ATAD3C* exon positions are represented below according to their relative position within the KAlign alignment; this includes alignment gaps. The 398 bp region of 100% sequence identity is marked in yellow.

(B) Reference arrangement of the ATAD3 cluster showing the exon structures of *ATAD3C* (purple), *ATAD3B* (orange), and *ATAD3A* (green). The duplicated region is highlighted in red.

(C) The reference arrangement of the *ATAD3* cluster above the predicted configuration following duplication.

(D) The exon structure of the *ATAD3A-C* fusion gene, with exons 1–11 derived from ATAD3A (green) and exons 12–16 derived from *ATAD3C* (purple). The ATPase domain is underlined (Asn347-Leu475; PFam PF00004), with the position of a key functional residue, Arg466, indicated by an arrow.

(E) Amino acid sequence of the ATPase domain of ATAD3A (top) and the predicted amino acids sequence of the ATAD3A-C fusion protein (bottom). The green residues are derived from *ATAD3A*, while the purple residues are derived from *ATAD3C*. A vertical bar (|) indicates an identical amino acid, a colon (:) indicates a strongly conservative amino acid change (score > 0.5 in PAM250 matrix), and a period (.) indicates a weakly conservative amino acid change (score = < 0.5 in PAM250 matrix). The sequences differ at seven positions.

non-alleleic homologous recombination (NAHR) between regions of high sequence homology in *ATAD3C* and *ATAD3A* (Figure 1A) and encompasses *ATAD3C* exons 8–12, *ATAD3B*, and *ATAD3A* exons 1–11 (Figures 1B, S1, and S2).

PCR and Sanger sequencing confirmed the presence of the duplications, which showed a 1.2 kb proband-specific amplicon (1.6 kb for subject four due to alternative primer design; data not shown). No PCR product was amplified in DNA derived from unaffected parents, consistent with a *de novo* event, and proband-parent relationships were confirmed for all case subjects during exome analysis. The 5′ end of the PCR amplicon was derived from *ATAD3A* exon 10, while the 3′ was derived from *ATAD3C* intron 7. The breakpoints of the duplication identified in subject four were found to differ from those identified in the other case subjects. The duplications are considered functionally equivalent as their protein products are predicted to be identical, differing at a single intronic nucleotide. These results are consistent with tandem duplication without inversion, described as NC_000001.11(GRCh38): 1456616_1524663dup (subjects 1–3 and 5) and NC_000001.11(GRCh38):1456890_1524937dup (subject 4). The duplications are predicted to maintain the copy-number of *ATAD3A* and *ATAD3C*, duplicate *ATAD3B*, and create a fusion gene, *ATAD3A-C*, composed of *ATAD3A* (Uniprot: Q9NVI7-2, residues 1–405) and *ATAD3C* (Uniprot: Q5T2N8-1, residues 231–411) (Figures 1B and 1C).

We performed multiple complementary *in silico* analyses to characterize the effect of the duplication. Multiple sequence alignment of *ATAD3A* (NC_000001.11 (GRCh38):1512151–1534687) and *ATAD3C* (NC_000001.11 (GRCh38):1449689–1470158) showed the genes have an overall sequence identity of approximately 56%. The duplications occur at a 673 bp region with near-complete sequence identity between *ATAD3A* and *ATAD3C* (Figure 1A). *In silico* splicing analysis of *ATAD3A-C* showed that the splice sites are maintained (Figure S3). Pairwise alignment of ATAD3A (GenBank: NM_001170535.2; Q9NVI7-2) and ATAD3A-C (Uniprot: Q9NVI7-2, residues 1–405, and Uniprot: Q5T2N8-1, residues 231–411) primary amino acid sequences showed that they are of identical length and differ at 29 residues (Figure S4). Seven of the variants (p.Val450Ile, p.Asn454Cys, p.Gln455His, p.Asp465Ala, p.Arg466Cys, p.Asn468Asp, and p.Glu469Val) lie within the ATPase domain (residues 348–474; Pfam: PF00004) (Figure 1D, underline; Figure 1E), while the remaining 22 are present outside of a known functional domain (p.Glu482Ala, p.Phe489Leu, p.Asp490Asn, p.Lys491Glu, p.Gln502Arg, p.Ser516Leu, p.Val518Ile, p.Gly527Cys, p.Glu529Lys, and p.Glu545Lys) or within a region of predicted intrinsic disorder (p.Thr556Ala, p.Arg557Cys, p.Ala561Phe, p.Lys568Met, p.Cys570Arg, p.Ala574Gly, p.Gly576Arg, p.Arg579Pro, p.Gly580Glu, p.Pro583Gln, p.Ser584Pro, and p.Pro585Ser). DeepLoc (v1.0) was used to predict the subcellular localization of

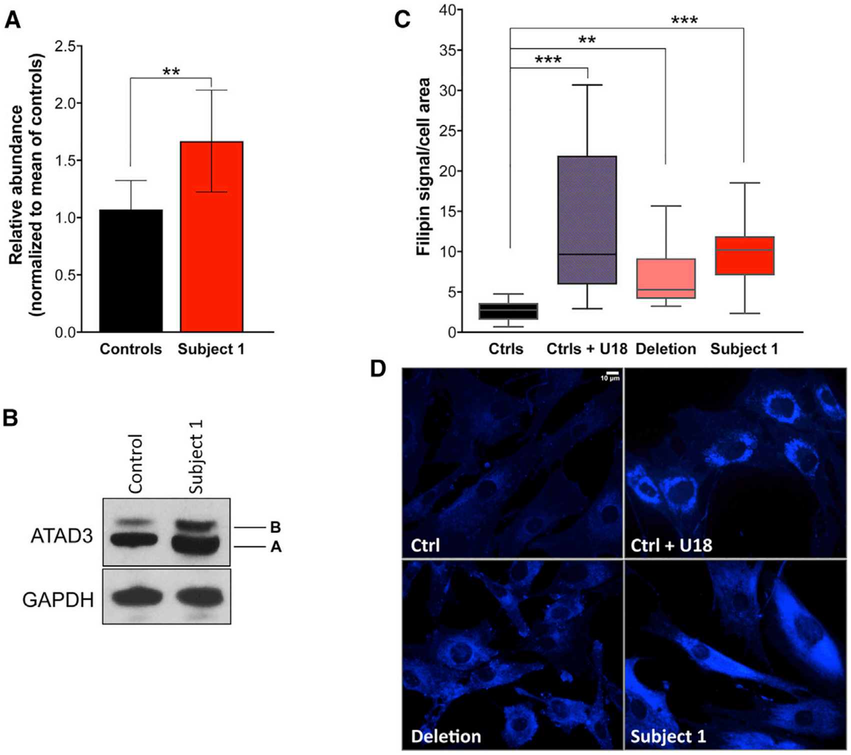

Figure 2. Elevated ATAD3 and Free Cholesterol Levels in Fibroblasts Harboring the *ATAD3* Gene Cluster Duplication
(A) Level of ATAD3 in fibroblast of subject 1 compared to control subjects (Fiji ImageJ densitometric analysis). The data are the mean of n = 6 independent experiments using three different control cell lines. Error bars show 1 standard deviation (**$p < 0.01$; Welch's t test).
(B) A representative ATAD3 immunoblot using a pan-specific antibody in fibroblasts. Levels of GAPDH were used as indicators of protein loading. The increased signal of the upper band [B] is consistent with the duplication of *ATAD3B*. ATAD3A isoform 2 and the predicted ATAD3A-C fusion protein are of identical size; hence, the increased signal of the lower band [A] is consistent with the fusion gene being expressed and stable.
(C) Chart showing mean filipin signal of cells quantified by ImageJ. Subject 1: fibroblasts of an individual with the *ATAD3* gene cluster duplication; Deletion: fibroblasts of an individual with a biallelic *ATAD3* gene cluster deletion (see Desai et al.[2] for details); U18: U18666A is an inhibitor of cholesterol trafficking; Filipin is a fluorescent marker, which binds specifically to unesterified cholesterol. Data are the results of 8 independent experiments for subject 1 and control subject(s) and n = 6 for the "deletion." Error bars show 1 standard deviation (***$p < 0.001$; **$p \leq 0.01$; one-way ANOVA).
(D) Representative images of filipin-stained cells. Scale bar 10 μm.

ATAD3A and ATAD3A-C. The tool was able to correctly predict that ATAD3A is transported into the mitochondrial membrane. There was no change in this prediction for ATAD3A-C. Together, these analyses indicate that the fusion transcript is likely to be correctly transcribed and translated and maintain the signals necessary for native subcellular localization. We next modeled the composition of ATAD3 hexamers using a binomial distribution based on two copies of *ATAD3A* and one copy of *ATAD3A-C*. It is predicted that 8.8% of ATAD3 hexamers would be comprised solely of wild-type ATAD3A monomers, while 91.2% would contain at least one copy of the ATAD3A-C fusion protein (Figure S5).

To experimentally assess the predictions of the *in silico* analyses we amplified a ~1.8 kb product by reverse transcription PCR on RNA extracted from fibroblasts (subject 4), using a primer pair specific to *ATAD3A* and *ATAD3C*. Sanger sequencing of this product showed a sequence identical to the predicted *ATAD3A-C* transcript (Figure S6). We found that the 5′ region of the *ATAD3A-C* fusion transcript corresponds to that of *ATAD3A*, splicing isoform two. Western blotting showed that fibroblasts (subject 1) harboring the duplication had higher expression of *ATAD3*, compared to controls (Figures 2A and 2B). The upper of the two bands is where ATAD3B migrates and so the increased signal is attributed to the additional copy of *ATAD3B*. As *ATAD3A* is not fully duplicated, the increased signal of the lower band suggests that the *ATAD3A-C* protein product is expressed and stable. ATAD3 is an established mitochondrial protein,[7] and antibody labeling of ATAD3 in fibroblasts of subject 1 revealed a distribution similar to control cells and to the mitochondrial outer membrane protein TOMM20 (Figure S7). Therefore, both the duplicated *ATAD3B* and the *ATAD3A-C* fusion gene protein product appear to be targeted to the mitochondria.

Variants in *bor*, an *ATAD3A* homolog in *Drosophila melanogaster*, are associated with a reduction in the number of mitochondria and mitochondrial structural abnormalities[1] and bi-allelic *ATAD3* cluster deletions have been shown to cause mitochondrial structural abnormalities and impaired cholesterol metabolism in human fibroblasts.[2] Therefore, we assessed mitochondrial morphology and cholesterol levels in our cellular models. In subject 1-derived fibroblasts, free-unesterified cholesterol assessed by filipin staining was significantly higher than control subjects and was similar to cells with pronounced ATAD3 deficiency caused by bi-allelic *ATAD3* cluster deletions[2] (Figures 2C and 2D). Many fibroblasts (subject 1) showed aggregations of mitochondria, and swollen and rounded organelles (Figure 3A; circled). Nevertheless, cells with an extensive and interconnected mitochondrial network were also apparent (Figure 3A). Immuno-staining for DNA indicated that the swollen mitochondria contained accumulations of mtDNA (Figure 3B; arrows). These features are similar to those associated with *ATAD3* cluster

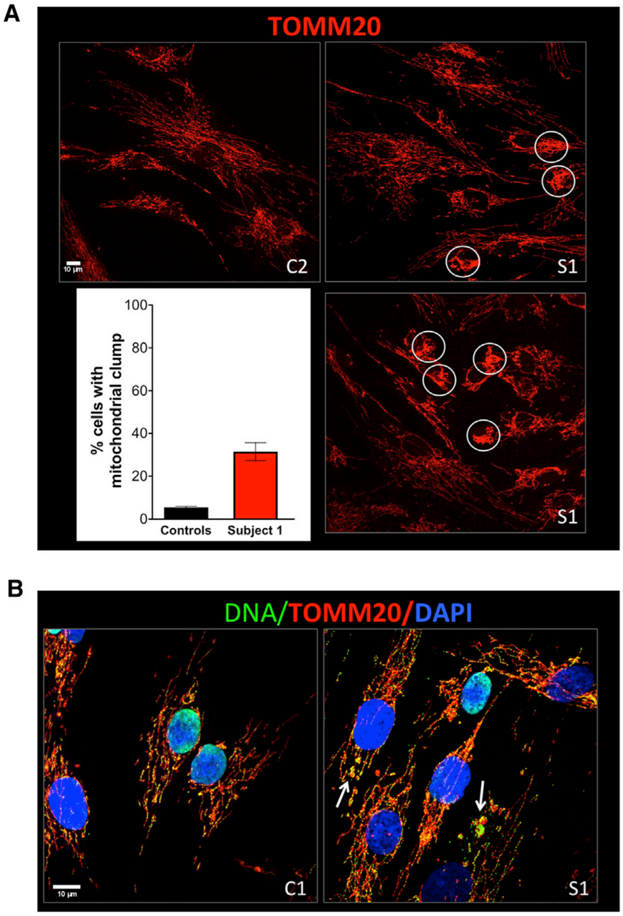

Figure 3. Abnormal Mitochondrial Morphology and mtDNA Organization in Cells with an *ATAD3* Gene Cluster Duplication
(A) Confocal images showing the mitochondria of control cell lines (C2) and fibroblasts from subject 1 (S1) labeled with an antibody to the outer mitochondrial membrane protein TOMM20 (red). Proportion of cells with clumped mitochondria for subject 1 versus 2 control subjects (n = 2 independent experiments, ≥50 cells per cell line, per experiment).
(B) Fibroblast cells from control subject (C1) and subject 1 (S1) labeled with an antibody against TOMM20 (red), a DNA antibody (green), and DAPI (blue); arrows indicate mtDNA aggregation. Scale bars 10 μm. Error bars show 1 standard deviation.

deletions;[2] we therefore infer that ATAD3A-C is dysfunctional and disrupts mitochondrial morphology and mtDNA organization and causes abnormalities in cellular cholesterol metabolism.

In addition to creating the ATAD3A-C fusion protein, the duplication creates an additional copy of *ATAD3B*. To determine whether this may have contributed to the subjects' phenotype, exome sequence data from all individuals in the Deciphering Developmental Disorders (DDD) cohort (n = 32,369) were evaluated for the presence of duplications affecting the *ATAD3* cluster. Excluding subject 5, who was identified in this cohort, 61 individuals were identified with likely monoallelic duplications intersecting the *ATAD3* cluster (size range of 67 kb–1.53 Mb), of which 48 affected only the *ATAD3* cluster. All duplications were found to fully encompass *ATAD3B* but did not intersect *ATAD3A*. Duplications were carried either by unaffected parents or probands whose clinical features were inconsistent with a probable metabolic disorder, were above the age of 1 year at their last clinical assessment, and were alive at the point of recruitment. Confirmation testing was not undertaken for the apparently benign duplications, and the precise breakpoints have not been determined. Nevertheless, the presence of multiple *ATAD3B* duplications in this study cohort suggests that the duplication of *ATAD3B* and increased *ATAD3B* gene dosage alone is not causative of this severe phenotype, but rather the NAHR-derived recombinant *ATAD3A-C* gene and novel protein product generated by the *de novo* mutational event.

We have identified two *de novo* duplications within the *ATAD3* cluster in five unrelated individuals whose clinical presentation suggested a metabolic disorder. *ATAD3* gene defects were recently recognized as a cause of human disease,[1–3] accounting for a growing number of phenotypes and cases. Dominant-negative *ATAD3A* missense variants have been reported in individuals affected with hypotonia, optic atrophy, axonal neuropathy, hypertrophic cardiomyopathy, and hereditary spastic paraplegia.[1,3] Bi-allelic *ATAD3* cluster deletions result in a more severe phenotype with pontocerebellar hypoplasia[2,14,15] and death in the majority of case subjects within the first week of life similar to bi-allelic *ATAD3A* deletions.[1] These case subjects with a monoallelic *ATAD3* gene cluster duplication extend the genotype spectrum of *ATAD3*-related disorders.

The phenotype of the neonates with *ATAD3* duplications shows overlap with the previously reported cases associated with pathogenic variation at this locus noting corneal clouding, cardiomyopathy, hypotonia, white matter changes, seizures, fetal akinesia, and contractures. All subjects with duplication died within 6 weeks of life. Although four neonates had low Apgar scores and required intensive clinical management from birth, subject 4 was born prematurely (33+3 weeks), achieved high Apgar scores, had a less severe perinatal course, and presented 3 weeks later with severe lactic acidosis (Table 1 and Supplemental Note). The subjects did not present with obvious signs of mitochondrial distress, and this study highlights the importance of considering mitochondrial genes even in atypical cases, such as these.

The *ATAD3A-C* fusion protein is uniquely associated with the severe neonatal phenotype and therefore is likely causal. It is expressed and stable (Figures 2A, 2B, and S6) and has the correct subcellular localization (Figure S7). The fusion protein differs from ATAD3A at 29 amino acid residues within the C-terminal region, including a highly conserved residue within the ATPase domain, p.Arg466Cys (Figure 1D; arrow and Figure 1E). The equivalent residue is conserved in all multimeric AAA-domain containing ATPases and functions as an arginine finger, a *trans*-acting residue that binds to the γ-phosphate of ATP in the neighboring monomer.[16] Multiple recurrent missense variants have been reported at the equivalent arginine finger residue, Arg499, in SPAST (Figure 4) and cause

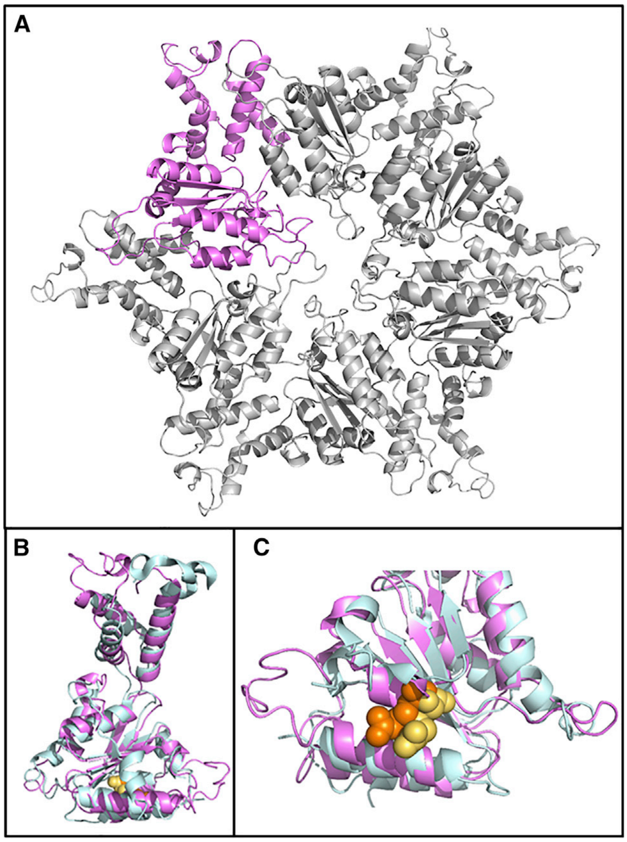

Figure 4. Protein Modeling of ATAD3 Hexamer and 3D Alignment against SPAST ATPase Domain
(A) Hexameric structure of ATAD3A ATPase domain (amino acids 348–474), modeled in SwissModel using PDB: 6f0x (*H. sapiens*, TRIP13) as a template. A single monomer is highlighted in violet.
(B) Single ATAD3A monomer (violet) aligned to *H. sapiens* SPAST ATPase domain (blue).
(C) The ATAD3A arginine finger, Arg466 (yellow) which is changed to a cysteine in the ATAD3A-C fusion gene, is overlaid with the SPAST arginine finger (Arg499; orange).

autosomal-dominant hereditary spastic paraplegia (SPG4 [MIM: 182601]).[17,18] These variants have been shown to result in the complete loss of SPAST ATPase activity,[19] leading to disease through a dominant-negative mechanism. We suggest that the *ATAD3* duplications described here act through the same mechanism: through incorporation of a non-functional monomer derived from the novel fusion protein into more than 90% of ATAD3 hexamers (Figure S5).

Our data suggest that the generation of the fusion protein causes this lethal neurological disorder through disruption of mitochondrial and cholesterol metabolism (Figures 2C, 2D, and 3). This reinforces the links between ATAD3, cholesterol, and mtDNA metabolism. Considering the majority of the cholesterol in mitochondrial membranes co-purifies with mtDNA,[20] and increasing or decreasing cholesterol availability markedly alters mtDNA organization,[2] then cholesterol dyshomeostasis evidently disrupts mtDNA metabolism. ATAD3 has links to cholesterol metabolism through partner proteins, TSPO, CYP11A1, and SPTLC.[6,8] ATAD3 also co-purifies with the mitochondrial protein synthesis machinery, mtDNA, and mitochondrial cholesterol,[7,8,20] and there is evidence that the mitochondrial nucleoprotein complexes are interlinked.[21,22] Hence perturbed cholesterol-containing micro-domains could be the common factor linking all the features associated with ATAD3 deficiency.

Copy number variants (CNVs) pose a practical challenge in genomic analysis, in both their detection and interpretation. Determining how to analyze and interpret rare CNVs which intersect common benign CNVs is not trivial. The high frequency of benign duplications seen in the *ATAD3* region coupled with high sequence homology of the three genes means that pathogenic duplications could potentially be missed. This study highlights the importance of systematic CNV analysis, particularly of genomic intervals prone to instability, where a clinical presentation is consistent with a monogenic disorder.

The high frequency at which this specific *ATAD3* duplication was identified within this cohort suggests that for all clinical suspicions of severe neonatal disorder of unknown origin, negative for known mitochondrial variants and mitochondrial nuclear genome panels, the *ATAD3* locus should be carefully evaluated for single nucleotide, copy-number, and structural variants.

Supplemental Data

Supplemental Data can be found online at https://doi.org/10.1016/j.ajhg.2020.01.007.

Acknowledgments

We acknowledge funding from Wellcome (200990). S.E. is a Wellcome Senior Investigator. U.F.P. is supported by a predoctoral fellowship from the Basque Government (PRE_2018_1_0253). M.M.O. is supported by a predoctoral fellowship from the University of the Basque Country (UPV/EHU, PIF 2018). I.J.H. is supported by the Carlos III Health Program (PI17/00380), and País Vasco Department of Health (2018111043; 2018222031). A.S. is supported by the UK Medical Research Council with a Senior Non-Clinical Fellowship (MC_PC_13029). T. Harel is supported by the Israel Science Foundation grant 1663/17. W.H.Y. is supported by the National Institute of General Medical Sciences of the National Institutes of Health through grant 5 P20 GM103636-07. J.R.L. is supported by the US National Institute of Neurological Disorders and Stroke (R35NS105078), the National Institute of General Medical Sciences (R01GM106373), and the National Human Genome Research Institute and National Heart Lung and Blood Institute (NHGRI/NHBLI) to the Baylor-Hopkins Center for Mendelian Genomics (BHCMG, UM1 HG006542). R.W.T. is supported by the Wellcome Centre for Mitochondrial Research (203105/Z/16/Z), the Medical Research Council (MRC) International Centre for Genomic Medicine in Neuromuscular Disease, Mitochondrial Disease Patient Cohort (UK) (G0800674), the UK NIHR Biomedical Research Centre for Aging and Age-related disease award to the Newcastle

upon Tyne Foundation Hospitals NHS Trust, the MRC/EPSRC Molecular Pathology Node, The Lily Foundation, and the UK NHS Highly Specialised Service for Rare Mitochondrial Disorders of Adults and Children. The DDD study presents independent research commissioned by the Health Innovation Challenge Fund (grant number HICF-1009-003). This study makes use of DECIPHER, which is funded by Wellcome. See Nature PMID: 25533962 or https://www.ddduk.org/access.html for full acknowledgment.

Declaration of Interests

Baylor College of Medicine (BCM) and Miraca Holdings have formed a joint venture with shared ownership and governance of Baylor Genetics (BG), which performs clinical microarray analysis and clinical exome sequencing. J.R.L. serves on the Scientific Advisory Board of BG. J.R.L. has stock ownership in 23andMe, is a paid consultant for Regeneron Pharmaceuticals, has stock options in Lasergen, and is a co-inventor on multiple United States and European patents related to molecular diagnostics for inherited neuropathies, eye diseases, and bacterial genomic fingerprinting. The other authors declare no competing interests.

Received: September 2, 2019
Accepted: January 10, 2020
Published: January 30, 2020

Web Resources

CBS DeepLoc, http://www.cbs.dtu.dk/services/DeepLoc/
DECIPHER, https://decipher.sanger.ac.uk/
EBI KAlign, https://www.ebi.ac.uk/Tools/msa/kalign/
EBI MUSCLE, https://www.ebi.ac.uk/Tools/msa/muscle/
Ensembl Genome Browser, http://www.ensembl.org/index.html
OMIM, https://www.omim.org/
Swiss-Model, https://swissmodel.expasy.org/
Uniprot, https://www.uniprot.org/

References

1. Harel, T., Yoon, W.H., Garone, C., Gu, S., Coban-Akdemir, Z., Eldomery, M.K., Posey, J.E., Jhangiani, S.N., Rosenfeld, J.A., Cho, M.T., et al.; Baylor-Hopkins Center for Mendelian Genomics; and University of Washington Center for Mendelian Genomics (2016). Recurrent de novo and biallelic variation of ATAD3A, encoding a mitochondrial membrane protein, results in distinct neurological syndromes. Am. J. Hum. Genet. *99*, 831–845.
2. Desai, R., Frazier, A.E., Durigon, R., Patel, H., Jones, A.W., Dalla Rosa, I., Lake, N.J., Compton, A.G., Mountford, H.S., Tucker, E.J., et al. (2017). ATAD3 gene cluster deletions cause cerebellar dysfunction associated with altered mitochondrial DNA and cholesterol metabolism. Brain *140*, 1595–1610.
3. Cooper, H.M., Yang, Y., Ylikallio, E., Khairullin, R., Woldegebriel, R., Lin, K.-L., Euro, L., Palin, E., Wolf, A., Trokovic, R., et al. (2017). ATPase-deficient mitochondrial inner membrane protein ATAD3A disturbs mitochondrial dynamics in dominant hereditary spastic paraplegia. Hum. Mol. Genet. *26*, 1432–1443.
4. Baudier, J. (2018). ATAD3 proteins: brokers of a mitochondria-endoplasmic reticulum connection in mammalian cells. Biol. Rev. Camb. Philos. Soc. *93*, 827–844.
5. Gilquin, B., Taillebourg, E., Cherradi, N., Hubstenberger, A., Gay, O., Merle, N., Assard, N., Fauvarque, M.O., Tomohiro, S., Kuge, O., and Baudier, J. (2010). The AAA+ ATPase ATAD3A controls mitochondrial dynamics at the interface of the inner and outer membranes. Mol. Cell. Biol. *30*, 1984–1996.
6. Rone, M.B., Midzak, A.S., Issop, L., Rammouz, G., Jagannathan, S., Fan, J., Ye, X., Blonder, J., Veenstra, T., and Papadopoulos, V. (2012). Identification of a dynamic mitochondrial protein complex driving cholesterol import, trafficking, and metabolism to steroid hormones. Mol. Endocrinol. *26*, 1868–1882.
7. He, J., Mao, C.-C., Reyes, A., Sembongi, H., Di Re, M., Granycome, C., Clippingdale, A.B., Fearnley, I.M., Harbour, M., Robinson, A.J., et al. (2007). The AAA+ protein ATAD3 has displacement loop binding properties and is involved in mitochondrial nucleoid organization. J. Cell Biol. *176*, 141–146.
8. He, J., Cooper, H.M., Reyes, A., Di Re, M., Sembongi, H., Litwin, T.R., Gao, J., Neuman, K.C., Fearnley, I.M., Spinazzola, A., et al. (2012). Mitochondrial nucleoid interacting proteins support mitochondrial protein synthesis. Nucleic Acids Res. *40*, 6109–6121.
9. Zhao, Y., Sun, X., Hu, D., Prosdocimo, D.A., Hoppel, C., Jain, M.K., Ramachandran, R., and Qi, X. (2019). ATAD3A oligomerization causes neurodegeneration by coupling mitochondrial fragmentation and bioenergetics defects. Nat. Commun. *10*, 1371.
10. Murley, A., Lackner, L.L., Osman, C., West, M., Voeltz, G.K., Walter, P., and Nunnari, J. (2013). ER-associated mitochondrial division links the distribution of mitochondria and mitochondrial DNA in yeast. eLife *2*, e00422.
11. Lewis, S.C., Uchiyama, L.F., and Nunnari, J. (2016). ER-mitochondria contacts couple mtDNA synthesis with mitochondrial division in human cells. Science *353*, aaf5549.
12. Peralta, S., Goffart, S., Williams, S.L., Diaz, F., Garcia, S., Nissanka, N., Area-Gomez, E., Pohjoismäki, J., and Moraes, C.T. (2018). ATAD3 controls mitochondrial cristae structure in mouse muscle, influencing mtDNA replication and cholesterol levels. J. Cell Sci. *131*, jcs217075.
13. Issop, L., Fan, J., Lee, S., Rone, M.B., Basu, K., Mui, J., and Papadopoulos, V. (2015). Mitochondria-associated membrane formation in hormone-stimulated Leydig cell steroidogenesis: role of ATAD3. Endocrinology *156*, 334–345.
14. Peeters-Scholte, C.M.P.C.D., Adama van Scheltema, P.N., Klumper, F.J.C.M., Everwijn, S.M.P., Koopmans, M., Hoffer, M.J.V., Koopmann, T.T., Ruivenkamp, C.A.L., Steggerda, S.J., van der Knaap, M.S., and Santen, G.W.E. (2017). Genotype-phenotype correlation in ATAD3A deletions: not just of scientific relevance. Brain *140*, e66.
15. Frazier, A.E., Holt, I.J., Spinazzola, A., and Thorburn, D.R. (2017). Reply: Genotype-phenotype correlation in ATAD3A deletions: not just of scientific relevance. Brain *140*, e67.
16. Ogura, T., Whiteheart, S.W., and Wilkinson, A.J. (2004). Conserved arginine residues implicated in ATP hydrolysis, nucleotide-sensing, and inter-subunit interactions in AAA and AAA+ ATPases. J. Struct. Biol. *146*, 106–112.
17. Dong, E.-L., Wang, C., Wu, S., Lu, Y.-Q., Lin, X.-H., Su, H.-Z., Zhao, M., He, J., Ma, L.X., Wang, N., et al. (2018). Clinical

spectrum and genetic landscape for hereditary spastic paraplegias in China. Mol. Neurodegener. *13*, 36.
18. Hazan, J., Fonknechten, N., Mavel, D., Paternotte, C., Samson, D., Artiguenave, F., Davoine, C.S., Cruaud, C., Dürr, A., Wincker, P., et al. (1999). Spastin, a new AAA protein, is altered in the most frequent form of autosomal dominant spastic paraplegia. Nat. Genet. *23*, 296–303.
19. Evans, K.J., Gomes, E.R., Reisenweber, S.M., Gundersen, G.G., and Lauring, B.P. (2005). Linking axonal degeneration to microtubule remodeling by Spastin-mediated microtubule severing. J. Cell Biol. *168*, 599–606.
20. Gerhold, J.M., Cansiz-Arda, Ş., Lõhmus, M., Engberg, O., Reyes, A., van Rennes, H., Sanz, A., Holt, I.J., Cooper, H.M., and Spelbrink, J.N. (2015). Human Mitochondrial DNA-Protein Complexes Attach to a Cholesterol-Rich Membrane Structure. Sci. Rep. *5*, 15292.
21. Kehrein, K., Schilling, R., Möller-Hergt, B.V., Wurm, C.A., Jakobs, S., Lamkemeyer, T., Langer, T., and Ott, M. (2015). Organization of Mitochondrial Gene Expression in Two Distinct Ribosome-Containing Assemblies. Cell Rep. *10*, 843–853.
22. Durigon, R., Mitchell, A.L., Jones, A.W., Manole, A., Mennuni, M., Hirst, E.M., Houlden, H., Maragni, G., Lattante, S., Doronzio, P.N., et al. (2018). LETM1 couples mitochondrial DNA metabolism and nutrient preference. EMBO Mol. Med. *10*, e8550.

The American Journal of Human Genetics, Volume *106*

Supplemental Data

Recurrent *De Novo* NAHR Reciprocal Duplications in the *ATAD3* Gene Cluster Cause a Neurogenetic Trait with Perturbed Cholesterol and Mitochondrial Metabolism

Adam C. Gunning, Klaudia Strucinska, Mikel Muñoz Oreja, Andrew Parrish, Richard Caswell, Karen L. Stals, Romina Durigon, Karina Durlacher-Betzer, Mitchell H. Cunningham, Christopher M. Grochowski, Julia Baptista, Carolyn Tysoe, Emma Baple, Nayana Lahiri, Tessa Homfray, Ingrid Scurr, Catherine Armstrong, John Dean, Uxoa Fernandez Pelayo, Aleck W.E. Jones, Robert W. Taylor, Vinod K. Misra, Wan Hee Yoon, Caroline F. Wright, James R. Lupski, Antonella Spinazzola, Tamar Harel, Ian J. Holt, and Sian Ellard

Supplemental Materials

Supplemental note – Case reports

Figure S1. Exome read depth graph showing the *ATAD3* duplication

Figure S2. ArrayCGH copy-number findings for subject 4

Figure S3. *In silico* splicing prediction of the *ATAD3A-C* fusion gene

Figure S4. Amino acid sequence alignment of ATAD3A and ATAD3A-C

Figure S5. Predicted binomial distribution of ATAD3A and ATAD3A-C in ATAD3 hexameric structures

Figure S6. Nucleotide sequence alignment of a predicted *ATAD3A-C* fusion transcript and RT-PCR DNA product obtained from fibroblasts harboring the duplication

Figure S7. Antibody labelling of ATAD3 in fibroblasts reveals a similar distribution to control cells suggesting the ATAD3A-C fusion protein is targeted to mitochondria

Supplemental Methods

Supplemental References

Supplemental note – Case Reports

Subject 1 was a male child born at term with normal antenatal screening. Fetal akinesia was reported at 38+3 weeks gestation. At birth the child was bradycardic, with Apgars of 3. The child was resuscitated, intubated and transferred to a neonatal unit. He showed severe neonatal encephalopathy with seizures, bilateral corneal opacities, small penis and undescended testes. Renal ultrasound showed pelvicalyceal dilation. Echocardiogram showed poor left ventricular function and thickened right ventricle with abnormal trabeculation of the right ventricular apex. ArrayCGH, Prader-Willi syndrome and spinomuscular atrophy testing were normal. Extensive metabolic investigation showed increased excretion of fumarate, malate, 2-ketoglutarate, 3-methylglutaconate and 3-methylglutarate. Death occurred at three days of age.

Subject 2 was a female child born at 38 weeks with bilateral corneal opacities and bilateral single palmar creases. Antenatally, the neonate showed increased nuchal translucency but no evidence of trisomies 13, 18 or 21. There were poor Apgars at birth and the child was admitted to a neonatal intensive care unit. She developed hypoglycaemia and seizures; EEG analyses showed multifocal cerebral dysfunction; MRI brain showed simplified gyral patterning, temporal cysts and white matter changes. Cardiorespiratory arrest occurred at two weeks. Afterwards, cardiomyopathy was noted, acute renal failure and increasingly oedematous. Death occurred at six weeks of age.

Subject 3 was a male child born at term with normal antenatal screening. Apgars of 1 at birth. The child was grossly hydropic and was resuscitated, intubated ventilated and transferred to a neonatal intensive care unit where abnormal cranial ultrasound with encephalopathy was noted. Death occurred at 5 days of age. Post-mortem studies showed an enlarged dilated heart with endocardial fibrosis and focal myocyte necrosis and widespread hypoxic brain damage.

Subject 4 was a female child born at 33+3 weeks gestation who initially had mild feeding difficulties and hypotonia. She developed severe lactic acidosis at three weeks of life with worsening hypotonia requiring intubation. She was noted to have corneal clouding. She developed a significant pericardial effusion, persistent severe lactic acidosis, and death occurred at six weeks. During that time, a number of studies were done. An EEG showed diffuse cerebral dysfunction. A brain MRI demonstrated diffuse bilateral abnormal subcortical, periventricular, and deep white matter. Brain MR spectroscopy demonstrated decreased N-acetyl aspartate peak, markedly increased lactate peak, and small glutamine-glutamate peak. An echocardiogram showed concentric left ventricular hypertrophy with severely decreased function. Metabolic evaluations and a chromosomal microarray were nondiagnostic, except for urine organic acids, which showed markedly increased lactic acid, moderately increased 2-hydroxybutyric acid, fumaric acid, and 3-hydroxyisobutyric acids. A 202 gene Mitochondrial Genome Plus Mitochondrial Focused Nuclear Gene Panel (GeneDx, Gaithersburg, MD) revealed a heterozygous maternally inherited likely pathogenic variant in *DNM1L* (c.1588C>T; p.(Arg530Ter)). A second *DNM1L* variant was not identified and this maternally-inherited variant was therefore not considered causative. Electron transport chain enzyme studies on skin fibroblasts and very long chain fatty acids with plasmalogens on plasma and skin fibroblasts were normal.

Subject 5 was a male child born at term by Neville Barnes forceps due to a poor cardiotocography. He was hypotonic with bradycardia and had no respiratory effort at birth, but responded to IPPV and chest compression, and was crying with good respiratory effort at 15 minutes. Dense bilateral corneal clouding was noted, and there were mild limb contractures, and glandular hypospadias. He had generalized seizures at 4 days of life, treated with phenobarbitone. Respiratory effort was poor, and he was re-intubated. There may have been antenatal seizures, as daily episodes of fetal hiccoughing were described in the third trimester. An LP showed no evidence of infection, an EEG on

day 6 showed a burst suppression pattern, and a brain MRI demonstrated abnormal white matter and a mild reduction in brain volume. MR spectroscopy showed an increased lactate peak. Multiple renal cysts were noted on ultrasound. An echocardiogram showed a small ventricular septal defect. Although he had no further seizures, he remained hypotonic, with poor respiratory effort and had intermittent metabolic acidosis with raised lactate. At 3 weeks of age, he developed poor perfusion with deteriorating metabolic acidosis and echocardiography revealed an enlarged, dilated heart with wall thickening and a pericardial effusion. Despite inotropic support, diuretics, and respiratory support he did not improve. He died at 4 weeks, 5 days of age. Extensive metabolic investigation was undertaken in life, but was not conclusive. This included urine organic acids which were normal on day 3, but on day 22 there was increased lactate with moderate ketonuria along with a slight increase in malate and fumarate, possibly reflecting impaired mitochondrial function. At post mortem, cardiac muscle histochemistry showed a mosaic pattern of cytochrome c oxidase (COX) deficiency. A generally normal pattern was seen in skeletal muscle although there were some COX-deficient blood vessels. COX (mitochondrial complex IV) enzyme activity was deficient in homogenised heart muscle with sparing of complex II activity.

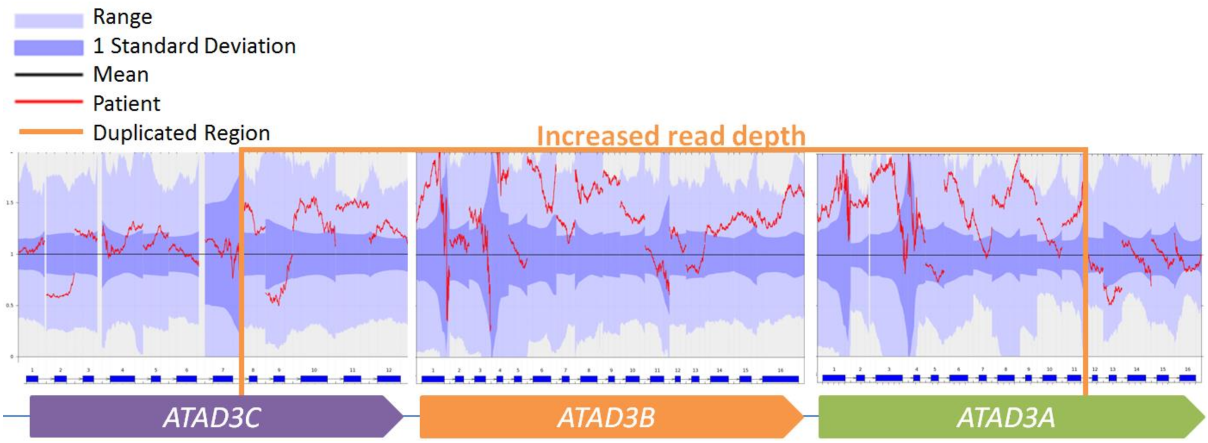

Figure S1. Exome read depth graph showing the *ATAD3* duplication. Normalized read-depth over the ATAD3 cluster for subject 1 (red line), compared to 1,634 samples previously analysed using the same capture library (Mean: Black Line; Range: light blue; 1 Standard Deviation: Dark Blue). The predicted extent of the duplication is shown in orange.

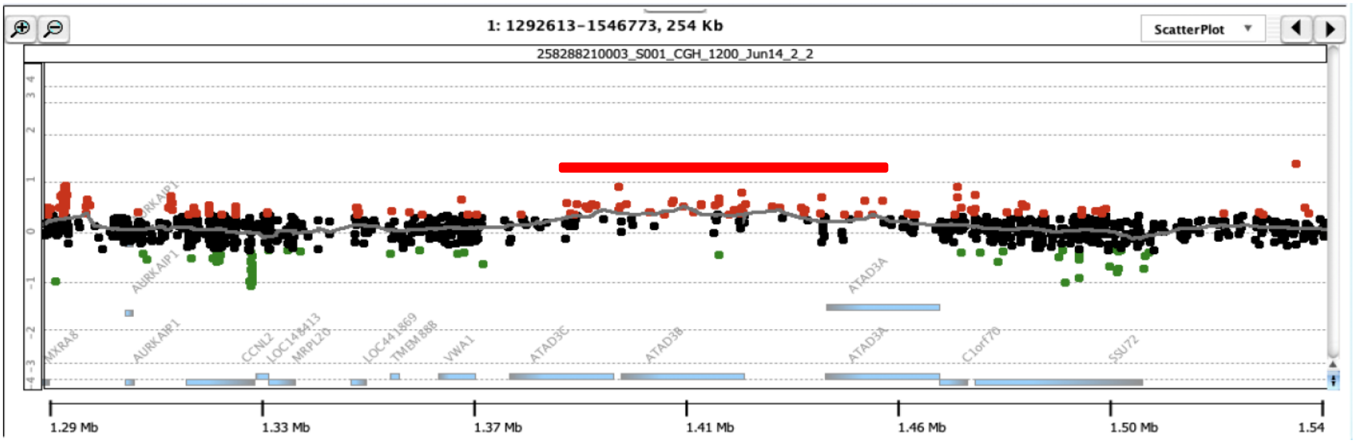

Figure S2. ArrayCGH copy-number findings for subject 4. The predicted duplicated region is represented by a red bar. There remain data-points within the normal range (black) indicating that the duplication is likely heterozygous.

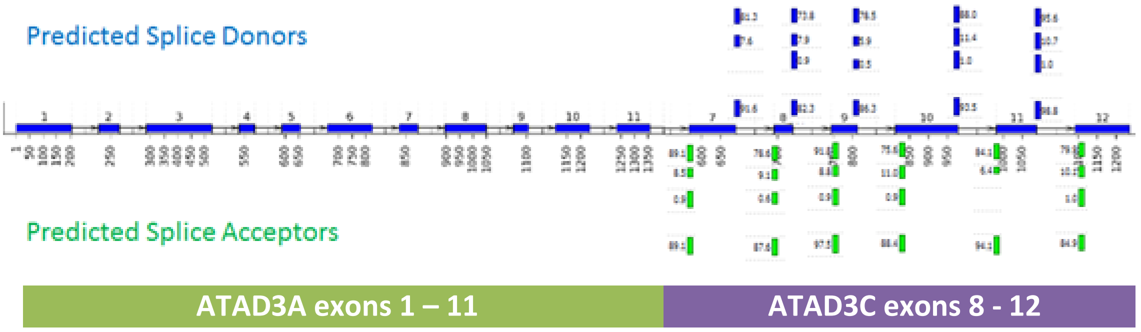

Figure S3. *In silico* splicing prediction of the *ATAD3A-C* fusion gene. Splicing prediction for the *ATAD3A-C* fusion gene, using the (1) SpliceSiteFinder-like, (2) MaxEntScan, (3) Human Splicing Finder algorithms. *ATAD3A* exon 11 and *ATAD3C* exon 7 are homologous. The plot shows that *ATAD3C* retains the splice sites necessary for correct mRNA processing.

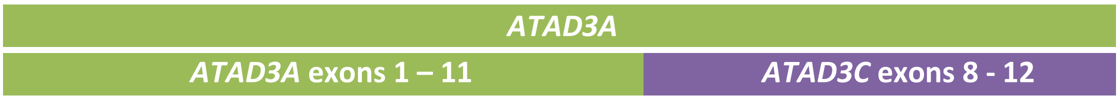


```
ATAD3A   1   MSWLFGINKGPKGEGAGPPPPLPPAQPGAEGGGDRGLGDRPAPKDKWSNFDPTGLERAAK
             ||||||||||||||||||||||||||||||||||||||||||||||||||||||||||||
Fusion   1   MSWLFGINKGPKGEGAGPPPPLPPAQPGAEGGGDRGLGDRPAPKDKWSNFDPTGLERAAK

ATAD3A   61  AARELEHSRYAKDALNLAQMQEQTLQLEQQSKLKEYEAAVEQLKSEQIRAQAEERRKTLS
             ||||||||||||||||||||||||||||||||||||||||||||||||||||||||||||
Fusion   61  AARELEHSRYAKDALNLAQMQEQTLQLEQQSKLKEYEAAVEQLKSEQIRAQAEERRKTLS

ATAD3A   121 EETRQHQARAQYQDKLARQRYEDQLKQQQLLNEENLRKQEESVQKQEAMRRATVEREMEL
             ||||||||||||||||||||||||||||||||||||||||||||||||||||||||||||
Fusion   121 EETRQHQARAQYQDKLARQRYEDQLKQQQLLNEENLRKQEESVQKQEAMRRATVEREMEL

ATAD3A   181 RHKNEMLRVEAEARARAKAERENADIIREQIRLKAAEHRQTVLESIRTAGTLFGEGFRAF
             ||||||||||||||||||||||||||||||||||||||||||||||||||||||||||||
Fusion   181 RHKNEMLRVEAEARARAKAERENADIIREQIRLKAAEHRQTVLESIRTAGTLFGEGFRAF

ATAD3A   241 VTDWDKVTATVAGLTLLAVGVYSAKNATLVAGRFIEARLGKPSLVRETSRITVLEALRHP
             ||||||||||||||||||||||||||||||||||||||||||||||||||||||||||||
Fusion   241 VTDWDKVTATVAGLTLLAVGVYSAKNATLVAGRFIEARLGKPSLVRETSRITVLEALRHP

ATAD3A   301 IQVSRRLLSRPQDALEGVVLSPSLEARVRDIAIATRNTKKNRSLYRNILMYGPPGTGKTL
             ||||||||||||||||||||||||||||||||||||||||||||||||||||||||||||
Fusion   301 IQVSRRLLSRPQDALEGVVLSPSLEARVRDIAIATRNTKKNRSLYRNILMYGPPGTGKTL

ATAD3A   361 FAKKLALHSGMDYAIMTGGDVAPMGREGVTAMHKLFDWANTSRRGLLLFVDEADAFLRKR
             ||||||||||||||||||||||||||||||||||||||||||||||||||||||||||||
Fusion   361 FAKKLALHSGMDYAIMTGGDVAPMGREGVTAMHKLFDWANTSRRGLLLFVDEADAFLRKR

ATAD3A   421 ATEKISEDLRATLNAFLYRTGQHSNKFMLVLASNQPEQFDWAINDRINEMVHFDLPGQEE
             |||||||||||||||||||||||||||||:|||..|||||||||..|:.|||||||||||
Fusion   421 ATEKISEDLRATLNAFLYRTGQHSNKFMLILASCHPEQFDWAINACIDVMVHFDLPGQEE

ATAD3A   481 RERLVRMYFDKYVLKPATEGKQRLKLAQFDYGRKCSEVARLTEGMSGREIAQLAVSWQAT
             |.||||||.::||||||||||:|||||||||||||.|:||||||||.|:|||||||||||
Fusion   481 RARLVRMYLNEYVLKPATEGKRRLKLAQFDYGRKCLEIARLTEGMSCRKIAQLAVSWQAT

ATAD3A   541 AYASEDGVLTEAMMDTRVQDAVQQHQQKMCWLKAEGPGRGDEPSPS 586
             ||||:||||||||||..|||.||||||.|.|||.|.||..||...|
Fusion   541 AYASKDGVLTEAMMDACVQDFVQQHQQMMRWLKGERPGPEDEQPSS 586
```

Figure S4. Amino acid sequence alignment of ATAD3A and ATAD3A-C. The predicted amino acid sequence of the ATAD3A-C fusion protein (green and purple) aligned against ATAD3A (black). A bar ('|') indicates an identical amino acid, a colon (':') indicates a strongly conservative amino acid change (score > 0.5 in the PAM250 matrix) and a period ('.') indicates a weakly conservative amino acid change (score ≤ 0.5 in the PAM250 matrix). The green residues are derived from the *ATAD3A* gene, while the purple residues are derived from *ATAD3C*. The sequences are of identical length and differ at 29 amino acid positions (highlighted in yellow). Two ATP-binding residues of known function are outlined in red; Asn454 and Arg466. Underlining in the ATAD3A sequence indicates residues of the conserved protein kinase domain [p.Ile348 – p.Asp474; PFam PF00004]. Residue numbering from [Q9NVI7-2 / NM_001170535.2].

Copies of fusion protein in hexamer	Proportion
0	8.8%
1	26.3%
2	32.9%
3	21.9%
4	8.2%
5	1.6%
6	0.1%

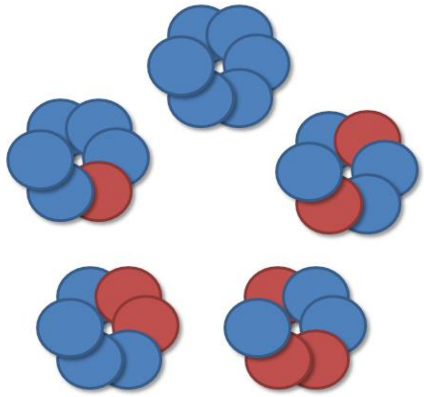

Figure S5. Predicted binomial distribution of ATAD3A and ATAD3A-C in ATAD3 hexameric structures. (Left) The predicted binomial distribution of ATAD3A-C monomers in a homo-hexameric ATAD3 quaternary structure, assuming a single copy of *ATAD3A-C* and two copies of *ATAD3A*. **(Right)** A cartoon showing the incorporation of non-functional ATAD3A-C monomers in the ATAD3 hexamer.

```
predicted      atgtcgtggctcttcggcattaacaagggccccaagggtgaaggcgcggggccgccgccg      60
RT-PCR         atGTCGTGGCTCTTCGGCATTAACAAGGGCCCCAAGGGTGAAGGCGCGGGGCCGCCGCCG      60
               ************************************************************

predicted      cctttgccgcccgcgcagcccggggccgagggcggcggggaccgcgggttgggagaccgg      120
RT-PCR         CCTTTGCCGCCCGCGCAGCCCGGGGCCGAGGGCGGCGGGGACCGCGGGTTGGGAGACCGG      120
               ************************************************************

predicted      ccggcgcccaaggacaaatggagcaacttcgacCCCACCGGCCTGGAGCGCGCCgccaag      180
RT-PCR         CCGGCGCCCAAGGACAAATGGAGCAACTTCGACCCCACCGGCCTGGAGCGCGCCGCCAAG      180
               ************************************************************

predicted      gcggcgcgcgagctggagcactcgcgttatgccaaggacgccctgaatctggcacagatg      240
RT-PCR         GCGGCGCGCGAGCTGGAGCACTCGCGTTATGCCAAGGACGCCCTGAATCTGGCACAGATG      240
               ************************************************************

predicted      caggagcagacgctgcagttggagcaacagtccaagctcaaagagtatgaggccgccgtg      300
RT-PCR         CAGGAGCAGACGCTGCAGTTGGAGCAACAGTCCAAGCTCAAAGAGTATGAGGCCGCCGTG      300
               ************************************************************

predicted      gagcagctcaagagcgagcagatccgggcgcaggctgaggagaggaggaagaccctgagc      360
RT-PCR         GAGCAGCTCAAGAGCGAGCAGATCCGGGCGCAGGCTGAGGAGAGGAGGAAGACCCTGAGC      360
               ************************************************************

predicted      gaggagacccggcagcaccAGGCCAGGGCCCAGTATCAAGacaagctggcccggcagcgc      420
RT-PCR         GAGGAGACCCGGCAGCACCAGGCCAGGGCCCAGTATCAAGACAAGCTGGCCCGGCAGCGC      420
               ************************************************************

predicted      tacgaggaccaactgaagcagcagcaacttctcaatgaggagaatttacggaagcaggag      480
RT-PCR         TACGAGGACCAACTGAAGCAGCAGCAACTTCTCAATGAGGAGAATTTACGGAAGCAGGAG      480
               ************************************************************

predicted      gagtccgtgcagaagcaggaagccatgcggcgagccaccgtggagcggGAGATGGAGCTG      540
RT-PCR         GAGTCCGTGCAGAAGCAGgaagccatgcggcgagccaccgtggagcgggagatggagctg      540
               ************************************************************

predicted      CGGCACAAGaatgagatgctgcgagtggaggccgaggcccgggcgcgcgccaaggccgag      600
RT-PCR         cggcacaagaatgagatgctgcgagtggaggccgaggcccgggcgcgcgccaaggccgag      600
               ************************************************************

predicted      cgggagaatgcagacatcatccgcgagcagatccgcctgaaggcggccgagcaccgtcag      660
RT-PCR         cgggagaatgcagacatcatccgcgagcagatccgcctgaaggcggccgagcaccgtcag      660
               ************************************************************

predicted      accgtcttggagtccatcaggacggctggcaccttgtttggggaaggattccgtgccttt      720
RT-PCR         accgtcttggagtccatcaggacggctggcaccttgtttggggaaggattccgtgccttt      720
               ************************************************************

predicted      gtgacagactgggacaaagtgacagccacggtggctgggctgacgctgctggctgttggG      780
RT-PCR         gtgacagactgggacaaagtgacagccacggtggctgggctgacgctgctggctgttggg      780
               ************************************************************

predicted      GTCTACTCAGCCAAGAATGCcacgcttgtcgccggccgcttcatcgaggctcggctgggg      840
RT-PCR         gtctactcagccaagaatgccacgcttgtcgccggccgcttcatcgaggctcggctgggg      840
               ************************************************************

predicted      aagccgtccctagtgagggagacgtcccgcatcacggtgcttgaggcgctgcggcacccc      900
RT-PCR         aagccgtccctagtgagggagacgtcccgcatcacggtgcttgaggcgctgcggcacccc      900
               ************************************************************

predicted      atccaggtcagccggcggctcctcagtcgaccccaggacgcgctggagggtgttgtgctc      960
RT-PCR         atccaggtcagccggcggctcctcagtcgaccccaggacgcgctggagggtgttgtgctc      960
               ************************************************************

predicted      agtcccagcctggaagcacgggtgcgcgacatcgccatagcaacaaggaacaccaagaag      1020
RT-PCR         agtcccagcctggaagcacgggtgcgcgacatcgccatagcaacaaggaacaccaagaag      1020
               ************************************************************

predicted      aaccgcagcctgtacaggaacatcctgatgtacgggccaccaGGCaccgggAAGacgctg      1080
RT-PCR         aaccgcagcctgtacaggaacatcctgatgtacgggccaccaggcaccgggaagacgctg      1080
               ************************************************************
```

```
predicted      tttgccaagaaactcgccctgcactcaggcatggactacgccatcatgacaggcggggac      1140
RT-PCR         tttgccaagaaactcgccctgcactcaggcatggactacgccatcatgacaggcggggac      1140
               ************************************************************

predicted      gtggcccccatggggcgggaaggcgtgaccgccatgcacAAGCTCTTTGACTGGGCCAAT      1200
RT-PCR         gtggcccccatggggcgggaaggcgtgaccgccatgcacaagctctttgactgggccaat      1200
               ************************************************************

predicted      accagccggcgcggcCTCCTGCTCTTTGTGGATGAAGCGGACGCCTTCCTTCGGAAGCGA      1260
RT-PCR         accagccggcgcggcctcctgctctttgtggatgaagcggacgccttccttcggaagcga      1260
               ************************************************************

predicted      GCCACTgagaagataagcgaggacctcagggccacactgaacgccttcctgtaccgcacg      1320
RT-PCR         gccactgagaagataagcgaggacctcagggccacactgaacgccTTCCTGTACCGCACG      1320
               ************************************************************

predicted      ggccagcacagcaacaaattcatgctgatcctggccagctgccaccccgagcagttcgac      1380
RT-PCR         GGCCAGCACAGCAACAAATTCATGCTGATCCTGGCCAGCTGCCACCCCGAGCAGTTCGAC      1380
               ************************************************************

predicted      tgggccatcaatgcctgcatcgacgtgatggtccacttcgacctgccagggcaggaggag      1440
RT-PCR         TGGGCCATCAATGCCTGCATCGACGTGATGGTCCACTTCGACCTGCCAGGGCAGGAGGAG      1440
               ************************************************************

predicted      cgggcgcgcctggtgagaatgtatcttaacgagtatgttcttaagccggccacagaagga      1500
RT-PCR         CGGGCGCGCCTGGTGAGAATGTATCTTAACGAGTATGTTCTTAAGCCGGCCACAGAAGGA      1500
               ************************************************************

predicted      aagcggcgtctgaagctggcccagtttgactacgggaggaagtgcttagagatcgctcgg      1560
RT-PCR         AAGCGGCGTCTGAAGCTGGCCCAGTTTGACTACGGGAGGAAGTGCTTAGAGATCGCTCGG      1560
               ************************************************************

predicted      ctgacagagggcatgtcatGCCGGAAGATCGCACAGCTGGccgtgtcctggcaggccacg      1620
RT-PCR         CTGACAGAGGGCATGTCATGCCGGAAGATCGCACAGCTGGCCGTGTCCTGGCAGGCCACG      1620
               ************************************************************

predicted      gcgtatgcctccaaggacggggtcctgaccgaggccatgatggacgcctgcgtgcaagac      1680
RT-PCR         GCGTATGCCTCCAAGGACGGGGTCCTGACCGAGGCCATGATGGACGCCTGCGTGCAAGAC      1680
               ************************************************************

predicted      tttgtccagcagcaccagcagatgatgcgctggctgaaggggggagaggcctgggcccgag      1740
RT-PCR         TTTGTCCAGCAGCACCAGCAGATGATGCGCTggctgaaggggggagaggcctgggcccgag      1740
               ************************************************************

predicted      gacgagcaaccctcatcctga     1761
RT-PCR         gacgagcaaccctcatcctga     1761
               *********************
```

Figure S6. Nucleotide sequence alignment of a predicted ATAD3A-C fusion transcript and RT-PCR DNA product obtained from fibroblasts harboring the duplication. The predicted nucleotide sequence of the *ATAD3A-C* fusion transcript aligned with that for a reverse transcription PCR (RT-PCR) product obtained from fibroblasts harboring the duplication (subject 4). The sequence obtained matches the predicted fusion transcript exactly.

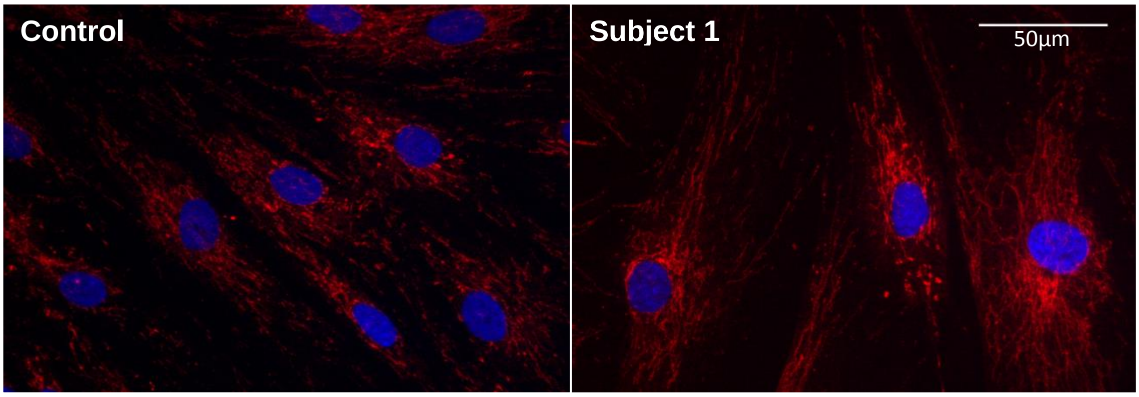

Figure S7. Antibody labelling of ATAD3 in fibroblasts reveals a similar distribution to control cells suggesting the ATAD3A-C fusion protein is targeted to mitochondria. Fibroblasts of control cells and subject 1 were labelled with anti-ATAD3 antibody (red) and DAPI (blue). Note the similar distribution to TOMM20 labeling [***Figure 3A***]. Co-staining cells with anti-ATAD3 and anti-TOMM20 confirmed the ATAD3 signal was restricted to the mitochondria (data not shown).

Supplemental Methods

Subjects

Cases 1-4 were referred for diagnostic trio exome sequencing due to a severe congenital developmental disorder. Case 5 was found through a manual analysis of the *ATAD3* cluster in the Deciphering Developmental Disorders (DDD) study cohort (4) for all participants with an age of death under one year (n=66). Informed consent for testing and publication was obtained from all participants.

Samples and genomic data

Sample collection, DNA extraction, arrayCGH, exome library preparation, trio exome sequencing, variant calling and annotation were performed as described (5). DNA was extracted from cultured skin fibroblasts from subjects one and four. Exome read-depth and arrayCGH data were assessed manually.

Confirmation of results by PCR and Sanger sequencing

Primers were designed using Primer3 (National Human Genome Research Institute, USA). For case four, a forward primer specific to *ATAD3A* intron 9 (Chr1(GRCh38):g.1523723-1523743) and a reverse primer specific to *ATAD3C* intron 8 (Chr1(GRCh38):g.1457228-1457250) were designed. For the other cases, the forward primer was located in *ATAD3A* exon 10 (NC_000001.11(GRCh38):g.1523875-1523893) and the reverse in *ATAD3C* intron 7 (NC_000001.11(GRCh38):g.1456957-1456977). Products were visualized by electrophoresis on a 3% agarose gel and sequenced bi-directionally.

Protein modelling

The crystal structure of the ATAD3 ATPase domain has not been solved; the ATPase domains of ATAD3A [Ensembl: ENST00000378756.8; Uniprot:Q9NVI7-2] and the predicted fusion protein [residues 1-405 of Q9NVI7-2, residues 231-411 of Q5T2N8-1, "ATAD3A-C"] were modelled using SWISS-MODEL (6). The highest scoring structure [PDB: 6f0x; H. sapiens TRIP13] was used as a template for modelling both structures. The models were visualized in PyMol.

***In silico* analyses**

In silico splicing analysis was performed on the splice junctions of ATAD3C exons 8-12, using the SpliceSiteFinder-like (1), MaxEntScan (2) and HSF (3) algorithms. The amino acid sequences of ATAD3A and ATAD3A-C were aligned using MUSCLE (7). The sequences were provided to DeepLoc (8) for analysis of the predicted subcellular localization. The genomic nucleotide sequences surrounding *ATAD3A* and *ATAD3C* were obtained from ENSEMBL and aligned using KAlign (9) to identify regions of homology.

Cell culture

Primary skin fibroblast cultures were obtained from subjects one and four, and healthy control individuals. Samples were confirmed free of mycoplasma based on the LookOut Mycoplasma PCR Detection Kit (Sigma). They were cultured in Dulbecco's Modified Eagle's Medium (DMEM, LifeTechnologies) supplemented with 10% fetal bovine serum (FBS, Hyclone/Sigma), 1% penicillin and streptomycin (PS, Life Technologies) at 37°C in a 5% CO_2 atmosphere.

Reverse transcription, and polymerase chain reaction

Total RNA was purified from fibroblasts obtained from subject 4 using TRIzol reagent (Life Technologies). cDNA was generated from 1 μg of total RNA using the SuperScript IV First-Strand Synthesis System (Life technologies). The ATAD3A-C fusion gene was amplified using a forward primer specific to ATAD3A (NC_000001.11(GRCh38):g.1512269-1512287) and a reverse primer specific to ATAD3C (NC_000001.11(GRCh38):g.1468505-1468530).

Western blot analysis, immunofluorescence and cell imaging

Protein fractionation, transfer and immuno-detection were performed as described (10). Cells were lysed on ice in phosphate-buffered saline (PBS), *n*-dodecyl-D-maltoside (DDM), 1X protease inhibitor cocktail (Roche), and phosphatase inhibitor cocktail (Abcam) and 50 Units Benzonase (Millipore). Protein concentration was determined by DC protein assay kit (Biorad). Protein samples were prepared in 1× Laemmli loading buffer, heated at 42°C for 15 minutes and resolved on SDS-PAGE gels (Novex, Thermofisher Scientific). After electrophoresis, proteins were transferred to polyvinylidene fluoride membranes (PVDF, Millipore). Membranes were blocked with 5% non-fat dry milk in PBS with 0.1% (v/v) Tween-20 (PBST) and incubated overnight at 4°C with the primary antibodies: anti-GAPDH (1:20 000, Abcam), anti-ATAD3 (1:50 000, gift from John Walker and Jiuya He).

Fibroblasts grown on coverslips were fixed with 4% paraformaldehyde in PBS for 15 minutes at 37°C. Cells were then washed three times for 5 minutes each with PBS before being permeabilized with 0.3% Triton X-100 in PBS containing 5% fetal bovine serum (PBSS) for 5 minutes at room temperature. After permeabilization, samples were washed and blocked with PBSS for 1 hour at room temperature and later incubated with the indicated primary antibodies: anti-DNA (1:200-250, Progen), anti-TOMM20 (abcam 1:100-500), anti-ATAD3A (1:100, Novusbio) at 4°C overnight. Following washes, cells were incubated for 1 hour at room temperature with secondary antibody, after which coverslips were mounted on glass slides over ProLong Gold Antifade Reagent. Unesterified cholesterol in fibroblasts was stained with filipin, using a cholesterol assay kit (Abcam), detected by wide-field fluorescence microscopy and quantified using ImageJ. Images of filipin stained cells were acquired with a Nikon eclipse 80i epifluorescence microscope, using the NIS elements software. Filipin signals (pixels/unit area) were quantified in Image J, using the Huang algorithm to define the area of the cells.

Supplemental References

1. Shapiro MB, Senapathy P. RNA splice junctions of different classes of eukaryotes: sequence statistics and functional implications in gene expression. Nucleic Acids Res. 1987 Sep 11;15(17):7155–7174.
2. Yeo G, Burge CB. Maximum entropy modeling of short sequence motifs with applications to RNA splicing signals. J Comput Biol. 2004;11(2-3):377–394.
3. Desmet F-O, Hamroun D, Lalande M, Collod-Béroud G, Claustres M, Béroud C. Human Splicing Finder: an online bioinformatics tool to predict splicing signals. Nucleic Acids Res. 2009 May;37(9):e67.
4. Wright CF, Fitzgerald TW, Jones WD, Clayton S, McRae JF, van Kogelenberg M, et al. Genetic diagnosis of developmental disorders in the DDD study: a scalable analysis of genome-wide research data. Lancet. 2015 Apr 4;385(9975):1305–1314.
5. Stals KL, Wakeling M, Baptista J, Caswell R, Parrish A, Rankin J, et al. Diagnosis of lethal or prenatal-onset autosomal recessive disorders by parental exome sequencing. Prenat Diagn. 2018;38(1):33–43.
6. Guex N, Peitsch MC. SWISS-MODEL and the Swiss-PdbViewer: an environment for comparative protein modeling. Electrophoresis. 1997 Dec;18(15):2714–2723.
7. Edgar RC. MUSCLE: multiple sequence alignment with high accuracy and high throughput. Nucleic Acids Res. 2004 Mar 19;32(5):1792–1797.
8. Almagro Armenteros JJ, Sønderby CK, Sønderby SK, Nielsen H, Winther O. DeepLoc: prediction of protein subcellular localization using deep learning. Bioinformatics. 2017 Nov 1;33(21):3387–3395.
9. Lassmann T, Frings O, Sonnhammer ELL. Kalign2: high-performance multiple alignment of protein and nucleotide sequences allowing external features. Nucleic Acids Res. 2009 Feb;37(3):858–865.
10. Dalla Rosa I, Durigon R, Pearce SF, Rorbach J, Hirst EMA, Vidoni S, et al. MPV17L2 is required for ribosome assembly in mitochondria. Nucleic Acids Res. 2014 Jul;42(13):8500–8515.
